# Biosynthesis and Emission of Stress-Induced Volatile Terpenes in Roots and Leaves of Switchgrass (*Panicum virgatum* L.)

**DOI:** 10.3389/fpls.2019.01144

**Published:** 2019-09-19

**Authors:** Andrew Muchlinski, Xinlu Chen, John T. Lovell, Tobias G. Köllner, Kyle A. Pelot, Philipp Zerbe, Meredith Ruggiero, LeMar Callaway, Suzanne Laliberte, Feng Chen, Dorothea Tholl

**Affiliations:** ^1^Department of Biological Sciences, Virginia Tech, Blacksburg, VA, United States; ^2^Department of Plant Sciences, University of Tennessee, Knoxville, TN, United States; ^3^Genome Sequencing Center, Hudson Alpha Institute for Biotechnology, Huntsville, AL, United States; ^4^Department of Biochemistry, Max Planck Institute for Chemical Ecology, Jena, Germany; ^5^Department of Plant Biology, University of California, Davis, Davis, CA, United States

**Keywords:** switchgrass, terpene synthase, volatile, herbivory, defense

## Abstract

Switchgrass (*Panicum virgatum* L.), a perennial C4 grass, represents an important species in natural and anthropogenic grasslands of North America. Its resilience to abiotic and biotic stress has made switchgrass a preferred bioenergy crop. However, little is known about the mechanisms of resistance of switchgrass against pathogens and herbivores. Volatile compounds such as terpenes have important activities in plant direct and indirect defense. Here, we show that switchgrass leaves emit blends of monoterpenes and sesquiterpenes upon feeding by the generalist insect herbivore *Spodoptera frugiperda* (fall armyworm) and in a systemic response to the treatment of roots with defense hormones. Belowground application of methyl jasmonate also induced the release of volatile terpenes from roots. To correlate the emission of terpenes with the expression and activity of their corresponding biosynthetic genes, we identified a gene family of 44 monoterpene and sesquiterpene synthases (mono- and sesqui-TPSs) of the type-a, type-b, type-g, and type-e subfamilies, of which 32 TPSs were found to be functionally active *in vitro*. The TPS genes are distributed over the K and N subgenomes with clusters occurring on several chromosomes. Synteny analysis revealed syntenic networks for approximately 30–40% of the switchgrass TPS genes in the genomes of *Panicum hallii*, *Setaria italica*, and *Sorghum bicolor*, suggesting shared TPS ancestry in the common progenitor of these grass lineages. Eighteen switchgrass TPS genes were substantially induced upon insect and hormone treatment and the enzymatic products of nine of these genes correlated with compounds of the induced volatile blends. In accordance with the emission of volatiles, TPS gene expression was induced systemically in response to belowground treatment, whereas this response was not observed upon aboveground feeding of *S. frugiperda.* Our results demonstrate complex above and belowground responses of induced volatile terpene metabolism in switchgrass and provide a framework for more detailed investigations of the function of terpenes in stress resistance in this monocot crop.

## Introduction

Switchgrass (*Panicum virgatum L.*, Poaceae) is a native warm-season C4 perennial grass common to natural and anthropogenic grasslands in North America. Characteristic of the Tallgrass Prairie, switchgrass is considered an important species for sustaining natural prairie biodiversity (Sanderson et al., 2006). Used mostly for forage since the 1950s, more intensive breeding of switchgrass began over 20 years ago to develop the species as an herbaceous model species for biofuel feedstock development (Casler et al., 2011). Major advantages for cultivating switchgrass are its resilience to extreme weather conditions, capability of growing on marginal soils, and a high cellulosic content (Vogel, 2004). Switchgrass also exhibits considerable resistance to pests and diseases (Parrish and Fike, 2005). With an increase in cultivation, growing interest has focused on elucidating the resistance mechanisms of switchgrass as well as engineering more resistant varieties. However, surprisingly little is still known about the modes of pathogen and pest defense in this species.

Plants deploy a biosynthetic and structurally diverse mosaic of specialized or secondary metabolites for chemical defense (Dudareva et al., 2004). Terpenes constitute the majority of such metabolites with important defensive activities. For instance, nonvolatile triterpenes are potent growth inhibitors of fungal pathogens (Osbourn, 1996). By contrast, low molecular weight 10-carbon monoterpenes and 15-carbon sesquiterpenes are emitted by plants as volatile compounds and serve important roles in direct defenses against pathogens and herbivores or function indirectly by the attraction of parasitoids or intra- and interplant priming (Turlings et al., 1990; Dicke, 1994; Kost and Heil, 2006; Köllner et al., 2008a; Huang et al., 2012; Vaughan et al., 2013; Erb et al., 2015).

The formation of terpenes in plants is catalyzed by enzymes of the terpene synthase superfamily (TPSs). TPS enzymes convert 10- and 15-carbon *cis*- or *trans*-isoprenyl diphosphates such as geranyl diphosphate (GDP), neryl diphosphate, farnesyl diphosphate [(*E*,*E*)-FDP or (*Z*,*Z*)-FDP] into monoterpenes or sesquiterpenes, respectively (Tholl and Lee, 2011). TPS genes often undergo species specific divergence and duplications resulting in terpene metabolic plasticity and adaptations (Pichersky and Gang, 2000). The structural diversity and biosynthetic evolution of terpene secondary metabolites have been studied extensively in crops including grasses such as maize, rice, and sorghum (Chen et al., 2011; Boutanaev et al., 2015; Block et al., 2019). Terpene-related defenses have been well described in these monocot crops and reveal diverse chemical mechanisms for resistance against above- and belowground stressors. For example, the sesquiterpene (*E*)-β-caryophyllene, one of the major volatile organic compounds (VOCs) released by maize leaves and roots, is involved in indirect defense by attracting parasitoids of herbivores and entomopathogenic nematodes (Turlings et al., 1990; Rasmann et al., 2005; Köllner et al., 2008a). Monoterpenes have also been implicated in defensive roles; for example, linalool confers resistance against rice bacterial blight caused by *Xanthomonas oryzae* (Taniguchi et al., 2014). More recently, a rice (*S*)-limonene synthase (*OsTPS19*) was shown to be involved in direct defense against the blast fungus *Magnaporthe oryzae* (Chen et al., 2018).

In contrast to these findings in highly domesticated grasses, the biosynthesis and dynamics of terpenes in switchgrass have not been fully investigated, in part because of its complex genetic background. Lowland ecotypes are allotetraploid (2*n* = 4*x* = 36), while upland cultivars are frequently octoploid (2*n* = 8*x* = 72). Recent transcriptional analysis of defense responses to green bug herbivory (*Schizaphis graminum*, Aphididae) in switchgrass leaves revealed a global transcriptional remodeling resulting in increased reactive oxygen species production and upregulation of genes with predicted terpene synthase function (Donze-Reiner et al., 2017). Moreover, the presence of a few triterpene saponins (C_30_) (Lee et al., 2009) and the synthesis of diterpenes (C_20_) related to abiotic stress have been described (Pelot et al., 2018). However, no prior studies have investigated the formation and function of volatile terpenes in this grass. Therefore, we sought to identify and characterize TPS genes from the switchgrass genome and correlate stress-induced terpene synthases with compound production in roots and leaves. Particular focus was placed on TPSs that were readily inducible when challenged with a generalist herbivore and the defense-related phytohormones methyl jasmonate (MeJA) and salicylic acid (SA) with the future goal to investigate these genes for their broad defensive functions against pathogens and herbivores. Results from this study provide further insight into the genetic organization of terpene metabolism in switchgrass and illustrate the metabolic potential of terpene-related defenses in perennial polyploid grasses.

## Materials and Methods

### Plant Materials

Seeds from the lowland allotetraploid switchgrass cv. Alamo were purchased from Bamert Seed Company (Muleshoe, TX) and used throughout this study. The seeds were sowed into potting substrate in 200-ml aluminum cans or 2.5″ pots and grown for 5 weeks at 26°C (16 h day) and 24°C (8 h night) in a Percival growth chamber. After germination, 15 seedlings were selected in each can or pot and grown for 5 weeks.

### Plant Treatments

Five-week-old seedlings were treated with larvae of *S. frugiperda* as described by Zhuang et al. (2012) with some modifications. Cans with 15 seedlings were each placed into a collection chamber, and 10 second instar larvae were released inside the chamber for overnight feeding. For treatment with MeJA and SA (Sigma-Aldrich), 25 ml of MeJA (0.1, 1, and 5 mM) or SA (5 mM) dissolved in ethanol were added per can or pot as a soil drench and left for 24 h, respectively. For physical wounding, a surgical scalpel was used to wound leaves and stems. Untreated plants and mock-treated plants (ethanol only) were used as controls. Three replicates were performed for each treatment.

### Volatile Collection and Identification

Volatiles emitted from leaves of the treated switchgrass and control plants placed in glass chambers were collected with an open headspace sampling system (Analytical Research Systems, Gainesville, FL, USA) in the light from 9:00 am to 1:00 pm. Fall armyworm (FAW) larvae were removed before volatile trapping. The volatiles were collected with volatile collection traps (Porapak-Q, http://www.volatilecollectiontrap.com/) and eluted with 100 µl methylene chloride containing 0.003% nonyl acetate (v/v). The collected volatiles were analyzed on a Shimadzu 17A gas chromatograph coupled to a Shimadzu QP5050A (http://www.shimadzu.com). Statistical analysis of leaf volatile data was done in R (v3.5.0) using ANOVA and *post-hoc* Tukey–Kramer honestly significant difference comparisons where alpha ≤0.05.

Root volatiles were analyzed by automated solid-phase microextraction (SPME, AOC-5000 Shimadzu) through adsorption in the headspace with a 100-µM polydimethylsiloxane (Supelco) SPME fiber and thermal desorption for gas chromatography–mass spectrometry (GC-MS) analysis. Root tissue (1 g fresh weight) was detached from plants and placed in a 20-ml screw-capped vial containing 2 ml distilled water and 20 ng of the volatile internal standard 1-bromodecane. The SPME fiber was placed into the headspace of the vial and incubated for 30 min at room temperature for volatile collection. Collected volatiles where thermally desorbed for 4 min and analyzed using a gas chromatograph (240°C injector port) coupled with a quadrupole mass spectrometer (GC-MS-QP2010S, Shimadzu). Extracts were separated with a 2:1 split on a 30 m × 0.25 mm i.d. × 0.25 μm film thickness Zebron capillary column (Phenomenex) using helium as the carrier gas (1.4 ml min^−1^ flowrate) and a temperature gradient of 5°C min^−1^ from 40°C (hold 2 min) to 220°C. Compound identification was based on similarity to library matches (NIST, Wiley), authentic standards (Sigma-Aldrich, (*E*)-β-caryophyllene, germacrene-D), and comparison to Opopanax essential oil (Floracopeia, δ-cadinene, α-humulene). Relative abundance was determined by normalization of the analyte peak area to the peak area of the internal standard and dividing by gram fresh weight.

### Identification of TPS Genes From the Switchgrass Genome and Phylogeny Reconstruction

Putative switchgrass TPS genes were retrieved from Phytozome (www.phytozome.jgi.doe.gov) through an annotation-based keyword search of genome versions v.1 and v.4. In addition, RNA-seq data kindly provided by the Noble Foundation (https://www.noble.org) for above- and belowground tissues were assembled *de novo* using Trinity (Grabherr et al., 2011). Assembled transcriptomes were queried with a representative switchgrass TPS sequence (*PvTPS01*) using the National Center for Biotechnology Information’s TBLASTX. Resulting BLAST hits were manually curated for putative functionality based on length and presence of the conserved aspartate rich motif (DDxxD) necessary for ionization of the prenyldiphosphate substrate (Class I TPSs). Class I and II diterpene synthases identified in this study were not further pursued based on previous reporting by Pelot et al. (2018). Gene models were refined further by comparing transcripts to genome sequences available in Phytozome. Putative N-terminal plastidic transit peptides were predicted using multiple sequence alignments and analysis of each sequence with the transit peptide prediction software ChloroP (Emanuelsson et al., 1999). Phylogeny reconstruction was based on protein sequence alignments, which were performed using MAFFT (Katoh et al., 2002). Maximum likelihood trees were then built from MAFFT alignments using PhyML (Guindon et al., 2010) with 500 bootstrap replicates as previously described (Pelot et al., 2018). Final phylogeny annotation and design were performed in Interactive Tree of Life (Letunic and Bork, 2007). Heat map analysis was based on publicly available expression data at http://www.phytozome.net/ following previously described methods (Pelot et al., 2018).

### Synteny Analysis and Identification of Orthologous TPS Genes

*P. virgatum* (v4.1), *Setaria italica* (v2.2), *Sorghum bicolor* (v3.1), and *P. hallii* var. *hallii* (v2.1) genome annotations were downloaded from phytozome (phytozome.jgi.doe.gov). Syntenic blocks were generated following Lovell et al. (2018) *via* the GENESPACE pipeline. Orthofinder was run on synteny-constrained BLASTp results to build orthologous gene networks.

### Gene Expression Analysis

Total RNA was isolated from switchgrass leaves and roots using the RNeasy Plant Mini Kit according to the manufacturer’s protocol (http://www.qiagen.com). Complementary DNA was synthesized using the GE Healthcare first-strand synthesis kit according to the manufacturer’s protocol (http://www.gelifesciences.com). Gene expression analysis was carried out using quantitative reverse transcription PCR (RT-PCR), which was described previously (Chen et al., 2018). Sequences of primers used for RT-qPCR are listed in **Supplementary Table 3**.

### Protein Expression in *E. coli* and Terpene Synthase Activity Assay

Full-length and truncated genes (predicted transit peptide removed) were synthesized and cloned (*Nde*I) into the pET-28b(+) prokaryotic expression vector. Constructs were transformed into *Escherichia coli* BL21-CodonPLus(DE3) cells (Stratagene) and grown at 37°C in 100 ml Luria–Bertani media supplemented with 50 µM kanamycin until an optical density at 600 nm (OD_600_) of 0.5–0.7. Protein production was then induced with 0.5 mM isopropyl 1-thio-ß-D-galactopyranoside, and cells were incubated with shaking at 18°C for 16 h. Recombinant protein extraction and partial purification were performed as described by Tholl et al. (2005), with the modification that N-terminal His-tags were implemented for partial purification. Enzyme reactions (125 µl total volume) were prepared in a 10-ml screw cap vial (Supelco) by combining partially purified protein with 20 mM MgCl_2_ and 60 µM commercially available prenyl diphosphate substrates GDP and (*E,E*)-FDP (Echelon Biosciences). Assay mixtures were incubated for 5 min at 30°C in the presence of a 100-µM polydimethylsiloxane fiber (Supelco). Collected volatiles were thermally desorbed for 4 min and analyzed using a gas chromatograph (240°C injector port) coupled with a quadrupole mass spectrometer (GC-MS-QP2010S, Shimadzu). Extracts were separated with a 5:1 split under the same conditions described above. Compound identification, in addition to those compounds described above, was based on similarity to library matches (NIST, Wiley, copaene, cycloisosativene, β-elemene, α-patchoulene, α-selinene, valencene), authentic standards (Sigma-Aldrich, borneol, 1,8-cineole, geraniol, limonene, linalool, α-pinene, sabinene, a-terpineol, α-terpinolene), and comparison to Opopanax oil [Floracopeia, β-bisabolene, (*E*)-γ-bisabolene, γ-curcumene, (*E*)-β-farnesene, sabinene, α-santalene].

## Results

### Emission of Volatile Terpenes From Leaves in Response to Insect Feeding

**Figure 1:**
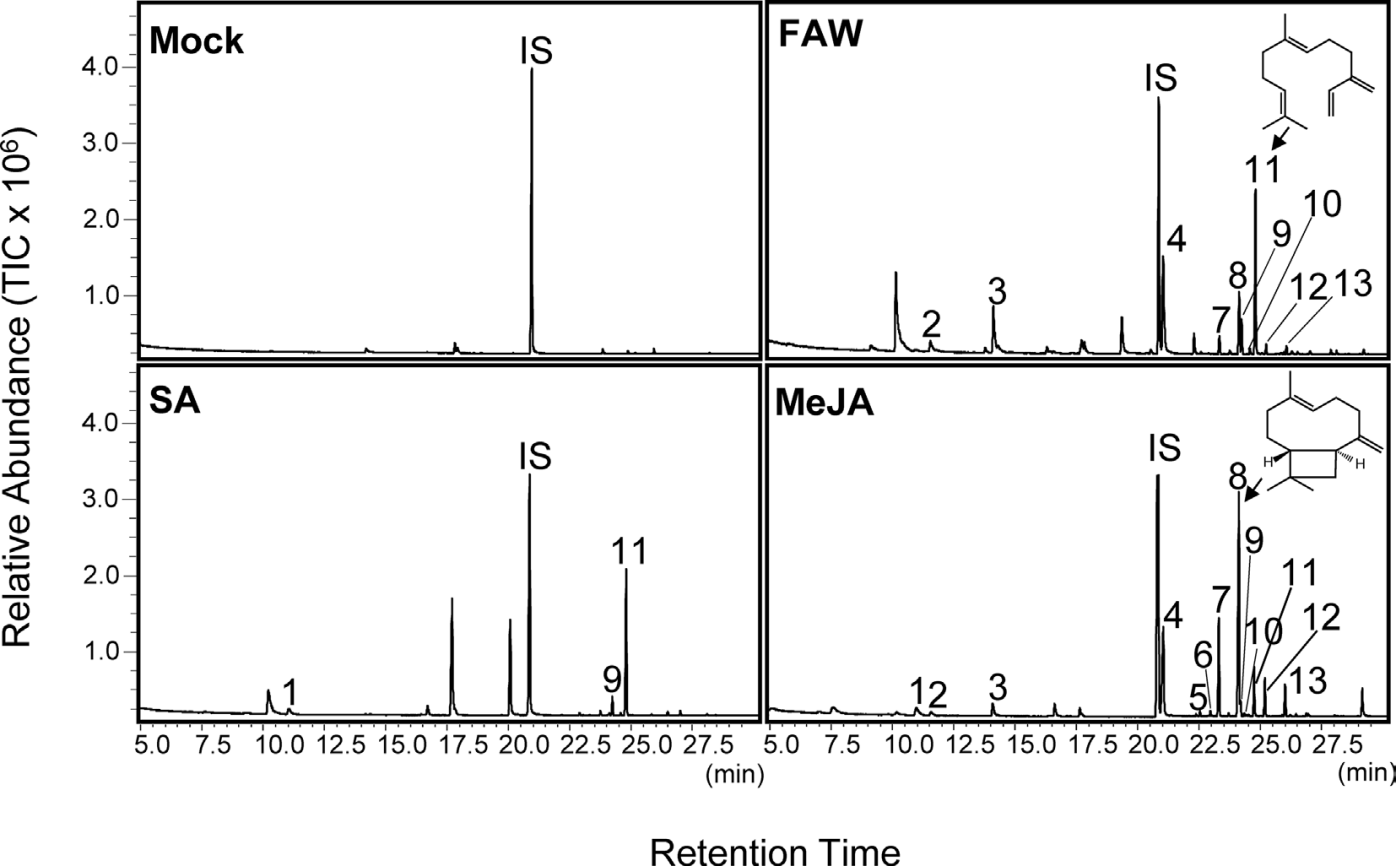
Gas chromatography–mass spectrometry (GC-MS) analysis of volatile emission from leaves of switchgrass (cv. Alamo) treated with fall armyworm (FAW), salicylic acid (SA), or methyl jasmonate (MeJA). Individual chromatograms are representative of samples analyzed in biological triplicates. Compound identification was based on similarity to library matches (NIST, Wiley) and comparisons to authentic standards. 1: limonene; 2: (*E*)-β-ocimene, 3: (*E*)-DMNT; 4: indole; 5: α-ylangene; 6: elemene isomer; 7: β-elemene; 8: (*E*)-β-caryophyllene; 9: α-bergamotene; 10: unidentified sesquiterpene; 11: (*E*)-β-farnesene; 12: α-humulene; and 13: β-copaene. IS: internal standard nonyl acetate.

To assess whether switchgrass leaves emit volatile compounds upon aboveground herbivory, emissions from switchgrass plants (cv. Alamo) damaged by larvae of *S. frugiperda* (FAW) were collected by open headspace sampling and analyzed by GC-MS. We found that FAW treatment induced the emission of nine terpene compounds, which were not detected in plants that only received physical wounding or remained untreated (**Figure 1** and **Supplementary Table 1**). Among the released compounds, the sesquiterpenes (*E*)-β-caryophyllene and (*E*)-β-farnesene were strongly induced by herbivore damage accounting for ∼17 and ∼26%, respectively, of the total volatile organic compound emission (**Figure 1** and **Supplementary Table 1**). Emission rates of (*E*)-β-caryophyllene were ∼500 ng/h g FW. Additional major compounds induced by FAW included the monoterpene (*E*)-β-ocimene, the homoterpene (*E*)-DMNT, and the sesquiterpenes β-elemene, α-bergamotene, α-humulene, and β-copaene (**Figure 1** and **Supplementary Table 1**).

### Emission of Volatile Terpenes From Roots and Leaves Upon Belowground Treatment With Methyl Jasmonate or Salicylic Acid

**Figure 2:**
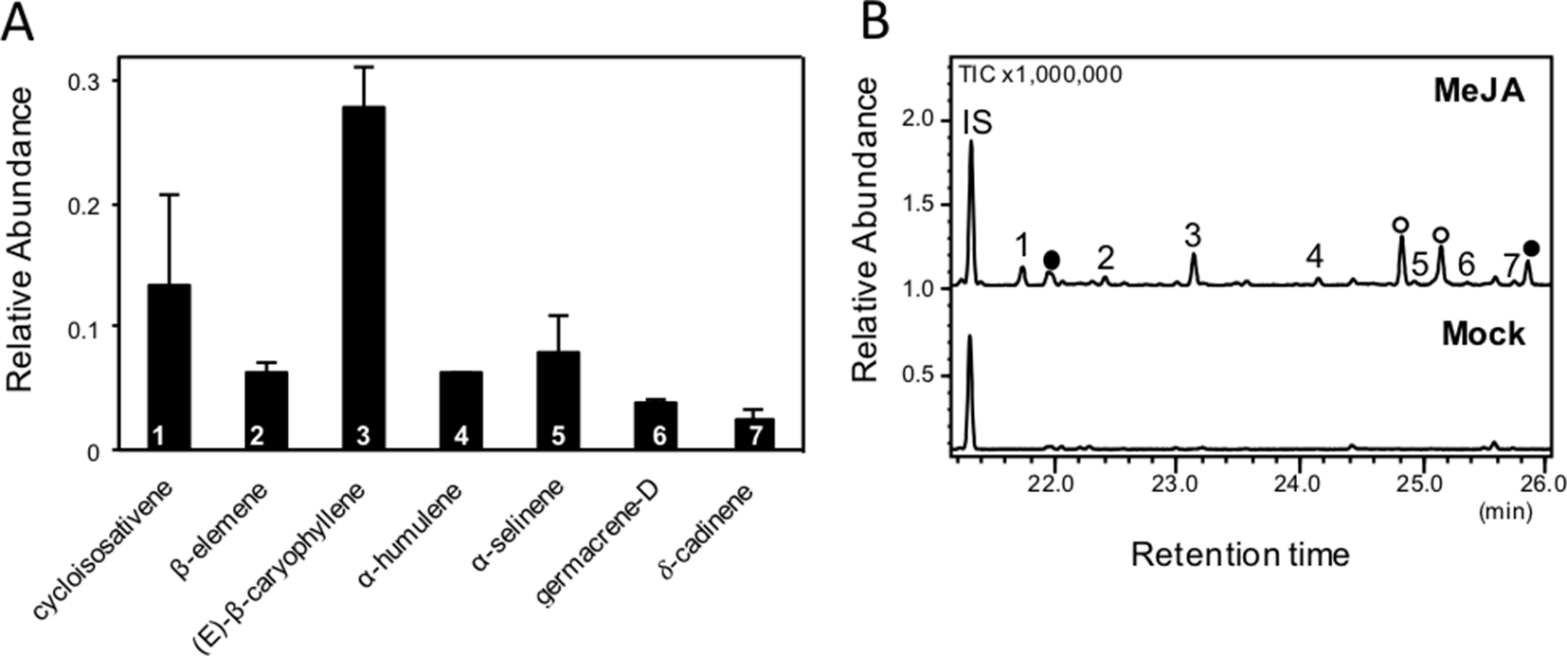
Solid-phase microextraction–gas chromatography–mass spectrometry (SPME-GC-MS) analysis of root sesquiterpene emission following methyl jasmonate (MeJA) treatment. Volatile compounds were analyzed in triplicate from detached pooled root material of 5-week-old plants. No sesquiterpenes were identified in the mock treatment. **(A)** Relative abundance of the detected sesquiterpenes. Samples were normalized to the internal standard and gram fresh weight. **(B)** Representative gas chromatograms of emitted volatiles in MeJA- and mock-treated roots. IS: internal standard 1-bromodecane; shaded circles indicate putative terpene compounds, and open circles indicate putative fatty acid derivatives. Compound identification was based on similarity to library matches (NIST, Wiley) and authentic standards.

We further determined whether emissions of volatile compounds from switchgrass roots could be induced by root treatment with phytohormones mimicking herbivory or pathogen infection. Different concentrations of MeJA were tested (0.1, 1, and 5 mM) by watering plants directly with each solution. Because of the volatility of MeJA, we expected that the compound diffused further into the substrate at a lower concentration. Volatiles were collected from detached roots using SPME and analyzed by GC-MS. Concentrations of 1 and 5 mM MeJA caused a similar relative release of sesquiterpene compounds from the root tissue (shown for 5 mM treatment; **Figure 2**), while no volatiles were induced upon treatment with 0.1 mM MeJA. Of the seven identified compounds, (*E*)-β-caryophyllene was the most abundant (∼43% of total), while cycloisosativene, β-elemene, α-humulene, α-selinene, germacrene D, and δ-cadinene were present at low levels. We also applied SA at a concentration of 5 mM; however, no release of sesquiterpenes was observed from root tissue. We further found two monoterpenoids, camphor and borneol, to be released from root tissue of untreated plants. Emissions of these compounds were reduced by MeJA and SA treatments, although this was not found to be statistically significant based on comparisons of the means (ANOVA, *p* > 0.05, **Supplementary Figure S1**).

We also tested whether a drench with MeJA and SA at 5 mM could induce volatile emissions in aboveground tissues. Treatment with MeJA strongly induced volatile emission from leaves compared to mock controls, with 13 compounds identified (**Figure 1** and **Supplementary Table 1**). Major induced compounds were (*E*)-β-caryophyllene and β-elemene accounting for ∼38 and ∼17%, respectively, of total volatile emissions (**Figure 1** and **Supplementary Table 1**). Emission rates of (*E*)-β-caryophyllene were approximately 1,500 ng/h g FW. Other minor compounds included limonene, (*E*)-β-ocimene, (*E*)-DMNT, α-ylangene, α-bergamotene, α-humulene, (*E*)-β-farnesene, and β-copaene (**Figure 1** and **Supplementary Table 1**). Two additional putative sesquiterpenes were also emitted; however, these compounds could not be further identified based on available standards. Treatment with SA induced the emission of four terpene compounds with (*E*)-β-farnesene accounting for 83% of total emissions. Trace amounts of limonene, (*E*)-β-caryophyllene, and α-bergamotene were detected, which were not observed in the untreated controls (**Figure 1** and **Supplementary Table 1**).

### Genome-Wide Identification of Putative Terpene Synthases in Switchgrass

Based on the inducible emission of diverse volatile terpenes from switchgrass roots and leaves, we sought to identify the TPS genes responsible for their formation. Following a genome-wide search of the switchgrass draft genome v.1, we originally identified 144 putative TPS gene models. Of these putative gene models, 108 were confirmed in the draft genome v.4, with 74 putative full-length mono-, sesqui-, and di-TPS genes identified. Manual sequence curation through multiple sequence alignments and comparison to genomic and transcriptomic data resulted in the identification of 44 putative full-length mono- and sesqui-TPS genes (**Table 1**, **Supplementary Table 2**, **Supplementary Figure S2**). Identified di-TPS genes (30 in total) were previously reported and therefore not included in this study (Pelot et al., 2018).

**Table 1:** Identified mono- and sesqui-terpene synthase (mono- and sesqui-TPS) gene models in the switchgrass (cv. “AP13”) genome in the order of chromosomal localization.

Locus ID	Designation	Protein Length	Genomic coordinates	Sub-family
Pavir.1KG024200	PvTPS28	520	Chr01K:2898821.2904002	tps-g
Pavir.1KG026700	PvTPS13	557	Chr01K:3056228.3059326	tps-g
Pavir.1KG213700	PvTPS83	607	Chr01K:30203541.30207022	tps-a
Pavir.1KG250000	PvTPS03	612	Chr01K:41080529.41082963	tps-a
Pavir.1KG359700	PvTPS33	574	Chr01K:60696889.60702775	tps-a
Pavir.1NG048300	PvTPS106	551	Chr01N:2491525.2496085	tps-g
Pavir.1NG047800	PvTPS27	552	Chr01N:2543130.2546026	tps-g
Pavir.1NG173900	PvTPS04	607	Chr01N:30384431.30393731	tps-a
Pavir.1NG245800	PvTPS26	583	Chr01N:56485952.56491693	tps-a
Pavir.2KG086400	PvTPS01	548	Chr02K:11130096.11132928	tps-a
Pavir.2KG150300	PvTPS14	552	Chr02K:19642302.19645362	tps-a
Pavir.2KG163100	PvTPS11	554	Chr02K:22460036.22464132	tps-a
Pavir.2NG122900	PvTPS85	515	Chr02N:18521205.18525163	tps-a
Pavir.2NG123000	PvTPS19	555	Chr02N:18533973.18537033	tps-a
Pavir.3KG098200	PvTPS36	592	Chr03K:8855955.8859293	tps-a
Pavir.3KG098400	PvTPS81	621	Chr03K:8870023.8873401	tps-a
Pavir.3KG102800	PvTPS08	616	Chr03K:9179806.9183931	tps-a
Pavir.5KG204300	PvTPS79	567	Chr05K:26556249.26559391	tps-a
Pavir.5KG258500	PvTPS09	491	Chr05K:38083739.38087664	tps-a
Pavir.6KG047500	PvTPS109	582	Chr06K:6526582.6531758	tps-a
Pavir.6KG066200	PvTPS17	549	Chr06K:9019105.9024173	tps-a
Pavir.6KG122500	PvTPS69	549	Chr06K:16365911.16369794	tps-a
Pavir.6KG141400	PvTPS10	259	Chr06K:23665655.23669023	tps-a
Pavir.6NG075100	PvTPS18	545	Chr06N:14008882.14012062	tps-a
Pavir.6NG075300	PvTPS16	626	Chr06N:14020511.14024883	tps-a
Pavir.6NG075700	PvTPS20	549	Chr06N:14043871.14051763	tps-a
Pavir.6NG192300	PvTPS94	545	Chr06N:36030001.36038500	tps-a
Pavir.6NG135600	PvTPS06	544	Chr06N:58557205.58561380	tps-a
Pavir.7NG405000	PvTPS15	618	Chr07N:63119161.63123381	tps-e
Pavir.8KG329300	PvTPS62	597	Chr08K:67429955.67433773	tps-a
Pavir.8NG101200	PvTPS101	549	Chr08N:15863965.15870973	tps-a
Pavir.9KG112900	PvTPS12	579	Chr09K:10384938.10389576	tps-b
Pavir.9KG454600	PvTPS56	570	Chr09K:66968728.66972114	tps-a
Pavir.9KG443500	PvTPS54	576	Chr09K:67815040.67822909	tps-a
Pavir.9KG442600	PvTPS55	552	Chr09K:67885565.67896951	tps-a
Pavir.9KG486800	PvTPS73	546	Chr09K:72969142.72975315	tps-b
Pavir.9NG517400	PvTPS50	551	Chr09N:86982070.86991194	tps-a
Pavir.9NG529400	PvTPS71	549	Chr09N:88336547.88343612	tps-a
Pavir.9NG574000	PvTPS05	560	Chr09N:92237810.92240737	tps-a
Pavir.9NG595500	PvTPS53	606	Chr09N:94252282.94256051	tps-a
Pavir.9NG595900	PvTPS104	467	Chr09N:94271551.94277158	tps-a
Pavir.9NG693900	PvTPS52	601	Chr09N:99983657.99989362	tps-b
Pavir.J27731	PvTPS07	502	contig312548:164.1302	tps-a
Pavir.J691000	PvTPS02	515	scaffold_732:11619.13941	tps-g

Genomic coordinates were determined based on draft genome data available in Phytozome (https://phytozome.jgi.doe.gov/).

**Figure 3:**
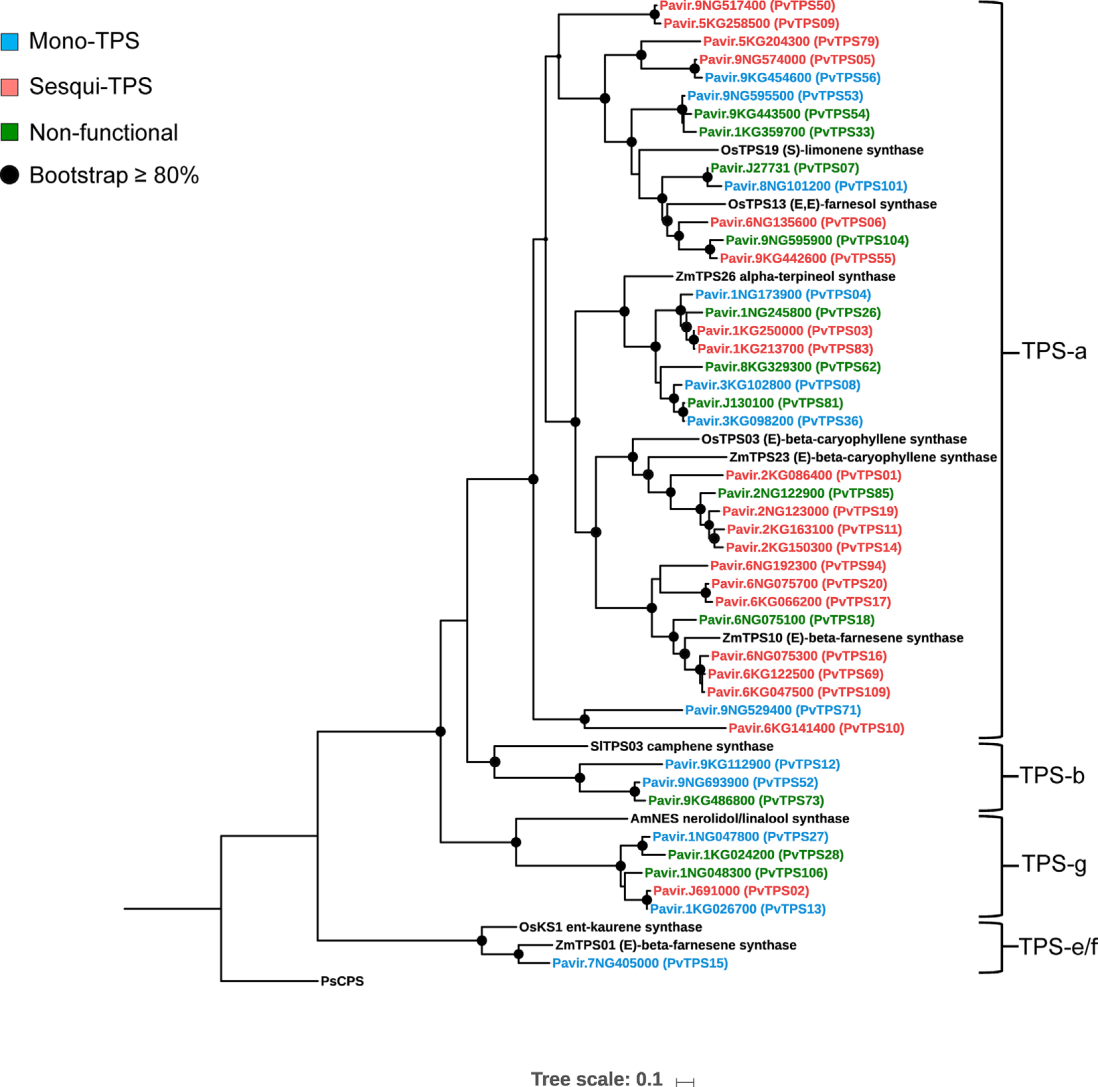
Maximum-likelihood phylogenetic tree based on multiple sequence alignment of 44 characterized mono- and sesqui-terpene synthases (mono- and sesqui-TPSs) in switchgrass and reference TPSs from maize, rice, sorghum, tomato, and snapdragon. Shaded circles represent branches with bootstrap support ≥80% (bootstrap replications = 500). The tree was rooted with the gymnosperm *Picea sitchensis ent*-CPP synthase (PsCPS).

Alignment and phylogenetic analysis of amino acid sequences from the mono- and sesqui-TPSs together with select TPSs from maize, rice, sorghum, tomato, and snapdragon showed that 35 members belong to the TPS type-a clade (**Figure 3**). In addition, five proteins aligned to the TPS-g subfamily and three clustered in the TPS-b subfamily. Only *Pv*TPS15 (TPS-e) was predicted to be involved in volatile formation outside of the TPS-a, TPS-b, and TPS-g subfamilies. Like in other plant TPS proteins, switchgrass TPSs of the TPS-a, TPS-b, TPS-e, and TPS-g subfamilies carry the conserved aspartate-rich “DDXXD” motif and the less conserved “NSE/DTE” motif in the C-terminal α-domain (Chen et al., 2011).

**Figure 4:**
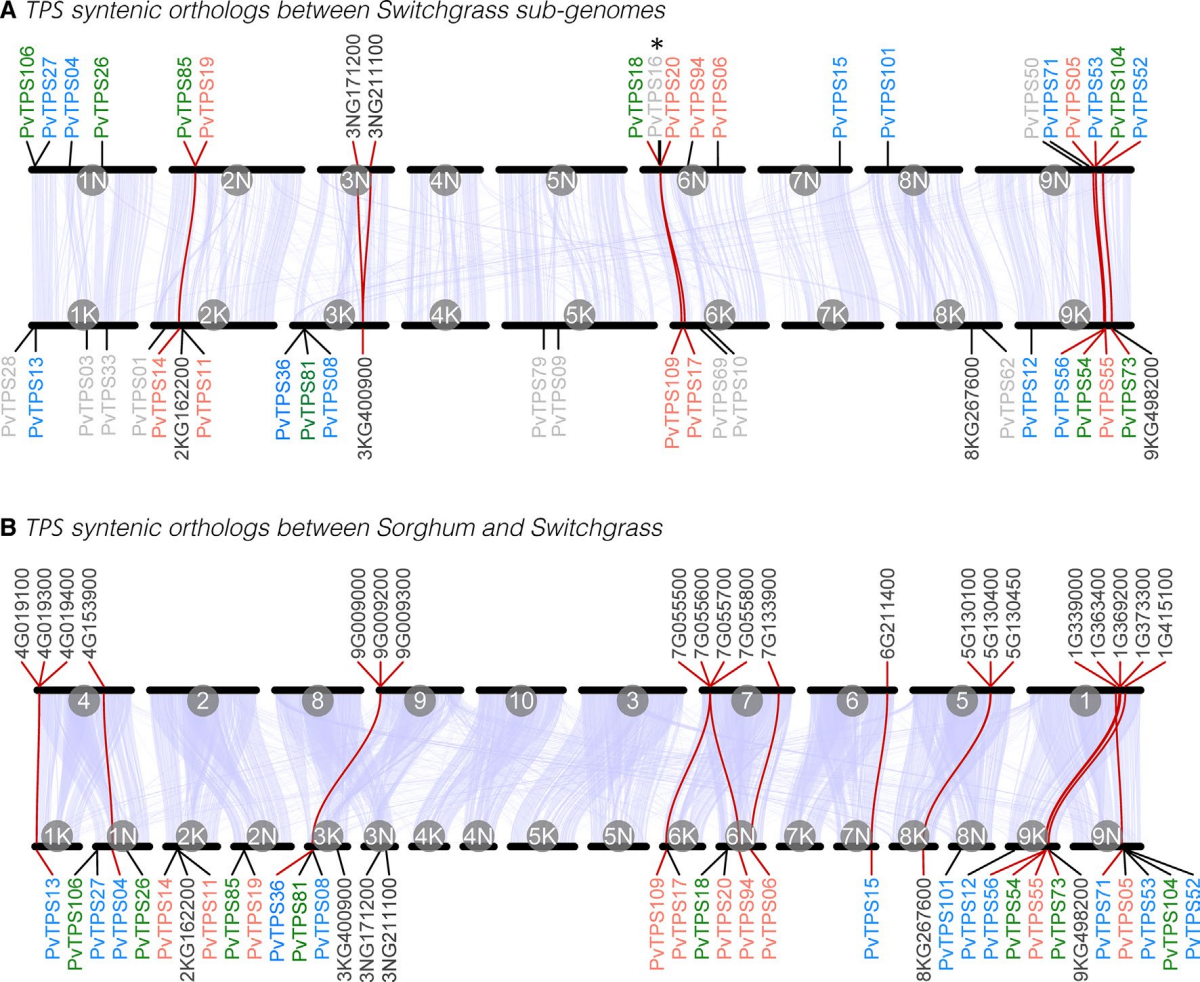
Synteny and orthology of terpene synthase (TPS) gene family members between *P. virgatum* subgenomes **(A)** and between *Panicum virgatum* and *Sorghum bicolor* **(B)**. In each plot, the genomic positions of chromosomes are plotted along the *x*-axis. The scale is independent for each genome and chromosomes are ordered to maximize synteny with *P. virgatum*. Chromosome IDs are printed on the inside of each line segment. Syntenic blocks between each pair of (sub)genomes are presented as light blue polygons. All characterized *P. virgatum* mono- and sesqui-TPS genes (labeled PvTPS, color code according to **Figure 3**) and annotated (functional or nonfunctional) TPS genes (labeled with the alpha-numeric Phytozome gene IDs) that occur in orthologous gene networks of any of the analyzed genomes (this figure and **Supplementary Figure S3**) are shown. Characterized *P. virgatum* mono- and sesqui-TPS genes that do not occur in orthologous gene networks are printed in light gray in **(A)**. The positions of genes are indicated by a straight line. A red line indicates that there is an ortholog in the alternative genome. *PvTPS16 has been only partially annotated in the *P. virgatum* v.4 genome.

When we examined the relative chromosomal position of the identified TPS genes, we found that 22 genes are distributed across the nine chromosomes in the switchgrass subgenome K with highest abundance of genes occurring on chromosomes 1K, 6K, and 9K (**Figure 4** and **Table 1**). In subgenome N, we identified the relative location of 20 genes with highest abundance on chromosomes 1N, 6N, and 9N (**Figure 4** and **Table 1**). Several genes are positioned in loose gene clusters throughout the genome (**Figure 4** and **Table 1**). The relative positions of *PvTPS02* and *PvTPS07* could not be determined based on incomplete genomic data (**Table 1**).

Investigation of syntenic orthologous genes between the two switchgrass subgenomes identified networks between 8 genes on subgenome K and 10 genes on subgenome N (including one putative mono- or di-TPS, 3NG211100, and two putative di-TPSs, 3KG400900 and 3NG171200) (**Figure 4**, **Supplementary Figure S3**, **Supplementary Table 2**). Comparisons between the genomes of switchgrass and sorghum showed that 13 switchgrass TPS loci have syntenic orthologs on 6 of the 10 sorghum chromosomes (**Figure 4**, **Supplementary Figure S3**, **Supplementary Table 2**). Several of these switchgrass TPS genes also occur in syntenic gene networks with genomes of the more closely related grasses *Setaria italica* and *Panicum hallii* suggesting conserved genomic regions in TPS gene evolution in these species.

### Biochemical Characterization of Monoterpene and Sesquiterpene Synthases From Switchgrass

**Figure 5:**
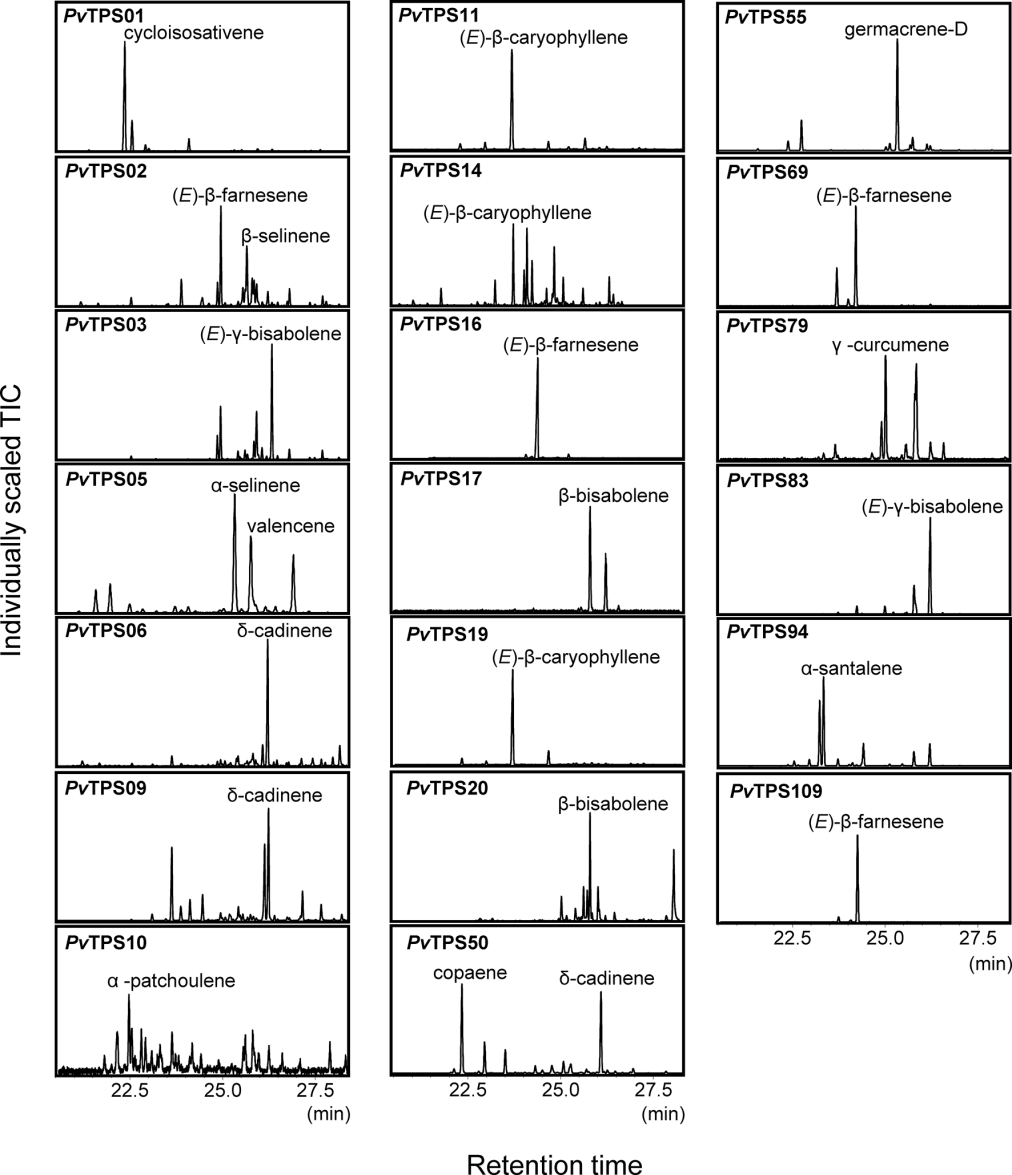
Solid-phase microextraction–gas chromatography–mass spectrometry (SPME-GC-MS) analysis of sesquiterpenes produced by recombinant terpene synthases (TPSs) with farnesyl diphosphate [(*E*,*E*)-FDP] as a substrate. Compound identification was based on similarity to library matches (NIST, Wiley), authentic standards and comparison to Opopanax oil (*Commiphora guidotti*). Unlabeled peaks represent minor enzymatically produced putative sesquiterpene compounds.

To determine the *in vitro* function of the 44 identified TPS genes, open reading frames were synthesized and cloned into the bacterial pET28b expression vector. The recombinant proteins were expressed in *E. coli* and protein lysates tested for TPS activity with GDP and (*E,E*)-FDP as substrates. We expected many TPSs in the subfamily-a (**Figure 3**) to function as sesqui-TPSs. Indeed, 19 recombinant TPS proteins in this family produced one or more sesquiterpene olefins, among them (*E*)-β-caryophyllene, (*E*)-β-farnesene, and other common plant sesquiterpenes (**Figures 3** and **5**). All of these proteins except *Pv*TPS83 did not carry a plastidial transit peptide, indicating that they are likely to function in the cytosolic compartment. *Pv*TPS02 was the only TPS protein found in the g-subfamily to exhibit sesquiterpene synthase activity *in vitro*. However, since a plastidial targeting sequence typical of subtype-g TPSs has been predicted for this protein, its function as a sesqui-TPS *in vivo* might be limited.

**Figure 6:**
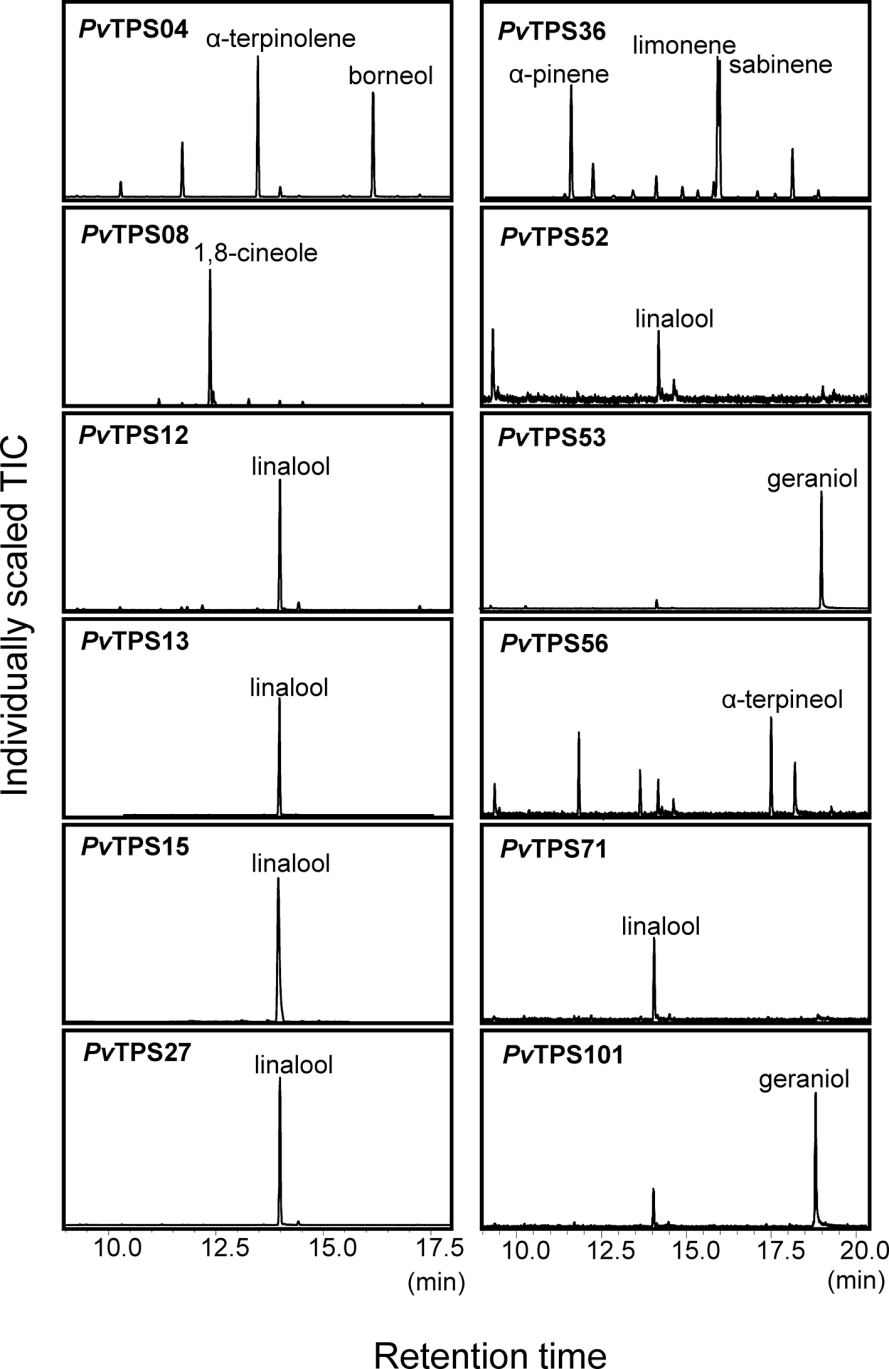
Solid-phase microextraction–gas chromatography–mass spectrometry (SPME-GC-MS) analysis of volatile monoterpenes produced by truncated recombinant terpene synthases (TPSs) with geranyl diphosphate (GDP) as a substrate. Compound identification was based on similarity to library matches (NIST, Wiley), authentic standards, and comparison to Opopanax oil (*Commiphora guidotti*).

Twelve TPS proteins distributed over the TPS-a, TPS-b, TPS-g, and TPS-e subfamilies functioned as monoterpene synthases *in vitro* (**Figures 3** and **6**). *Pv*TPS04 produced a mixture of monoterpenes from GDP with α-terpinolene and borneol as major products (**Figure 6**). *Pv*TPS36 and *Pv*TPS56 converted GDP into multiple volatile products with predominantly limonene and α-terpineol as the major products, respectively (**Figure 6**). The remaining enzymes produced either linalool (*Pv*TPS12, *Pv*TPS13, *Pv*TPS15, *Pv*TPS27, *Pv*TPS52, and *Pv*TPS71) or geraniol (*Pv*TPS53 and *Pv*TPS101) (**Figure 6**). *Pv*TPS13 and *Pv*TPS15 also converted (*E,E*)-FDP into nerolidol (**Supplementary Figure S4**); however, this activity might be limited *in vivo* because of the predicted plastidial localization of these proteins. On the contrary, no plastidial transit peptides were predicted for *Pv*TPS12, *Pv*TPS56, *Pv*TPS71, and *Pv*TPS101, which questions their function as monoterpene synthases *in vivo*.

Only trace amounts of compounds were detected for recombinant proteins encoded by *PvTPS07*, *PvTPS62*, *PvTPS81*, and *PvTPS106*. In addition, no substantial enzymatic activity was found for eight proteins (*Pv*TPS18, *Pv*TPS26, *Pv*TPS28, *Pv*TPS33, *Pv*TPS54, *Pv*TPS73, *Pv*TPS85, and *Pv*TPS104), which is in accordance with the presence of several deletions and/or insertions in the open reading frames of the corresponding genes (**Supplementary Figure S2**). Sequence truncations were furthermore found at the N- and C-terminus of the functionally active *Pv*TPS09 and *Pv*TPS02 proteins, respectively (**Supplementary Figure S2**).

### Expression Analysis of PvTPSs in Different Tissues and Upon Treatment With FAW, MeJA, and SA

**Figure 7:**
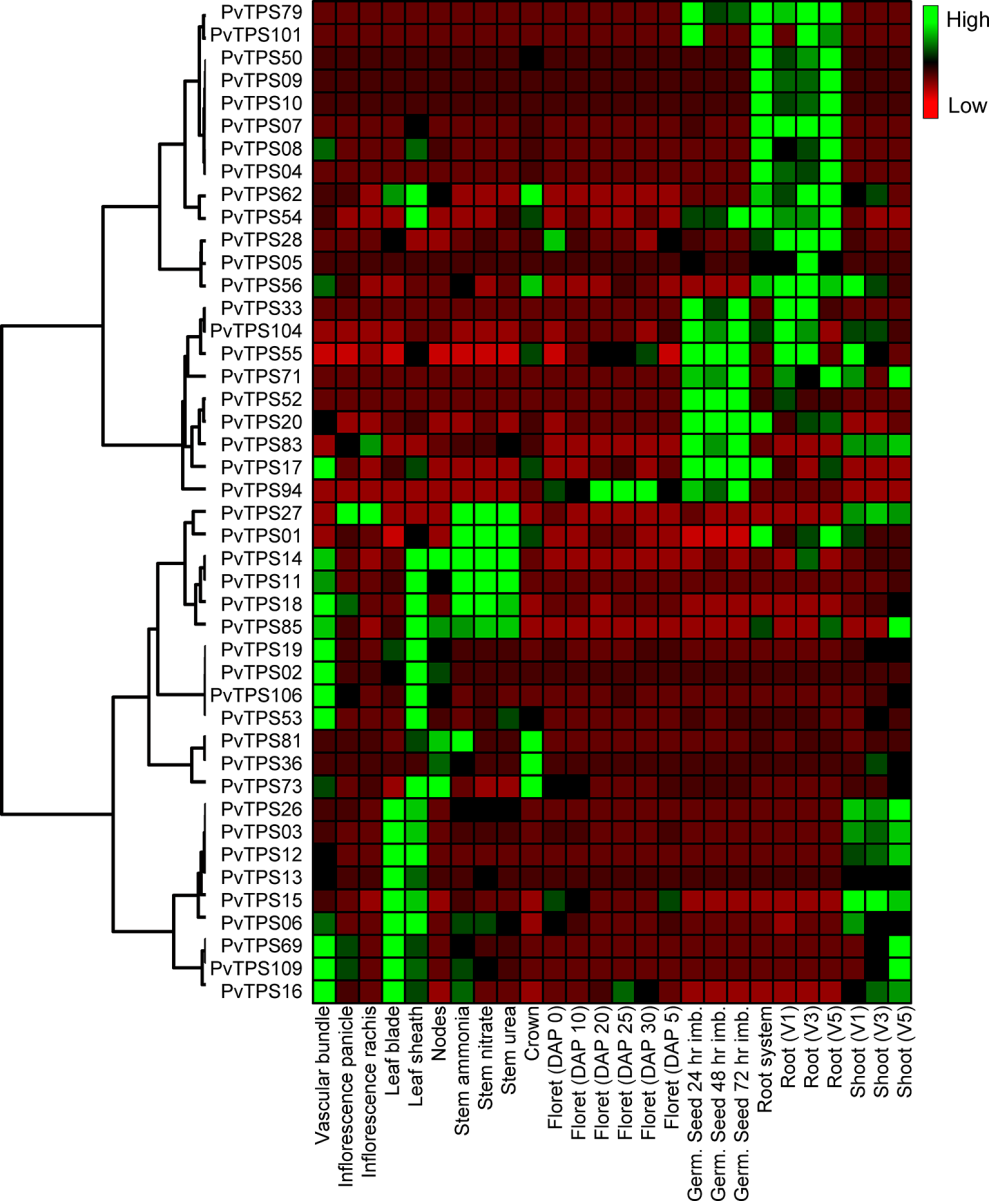
Hierarchical cluster analysis of terpene synthase (TPS) gene expression in different switchgrass (cv. “AP13”) tissues. The heat map compares relative transcript abundance for mono- and sesqui-TPS genes in fragments per kilobase of transcript per million mapped reads (FPKM) across 26 gene expression data sets (https://phytozome.jgi.doe.gov/).

Global expression patterns for all 44 TPS genes were analyzed by hierarchical cluster analysis based on publicly available data (https://phytozome.jgi.doe.gov/). We found specific patterns of transcript abundance in vascular tissue, leaf blade, and sheath tissues as well as roots and germinating seeds (**Figure 7**). Transcripts included those of the 12 genes that lack *in vitro* functional activity. There was little overlap in expression between above- and belowground tissues, indicating gene-specific adaptations in these tissues. Despite the observed transcriptional patterns, we were unable, with the exception of borneol, to detect volatile terpenes in leaves and roots of the Alamo cultivar under constitutive conditions.

**Figure 8:**
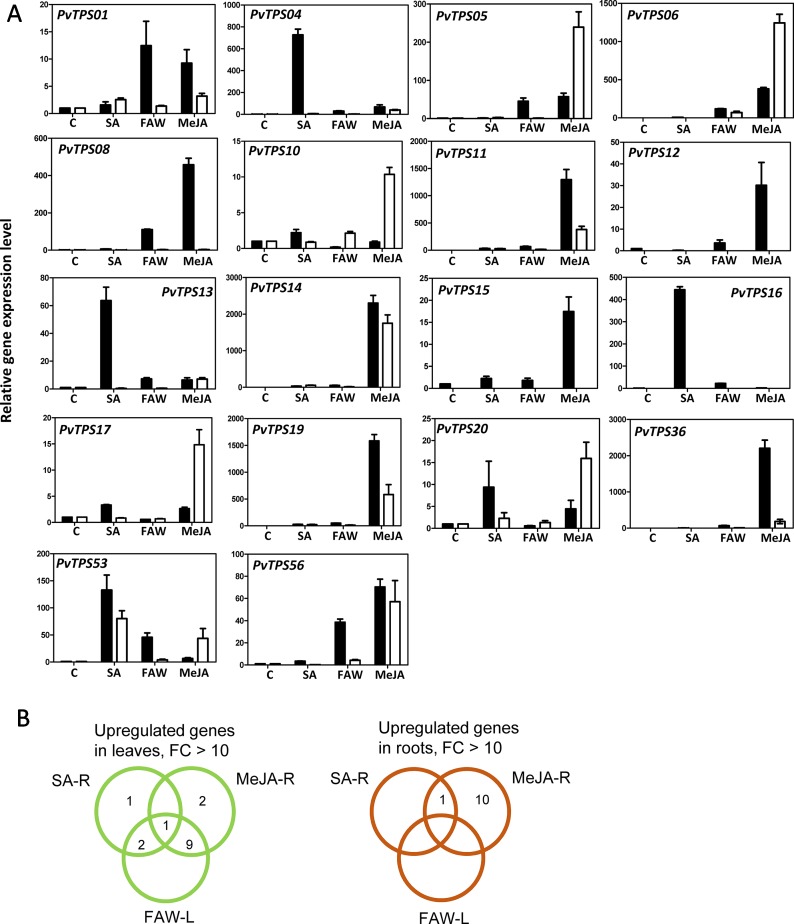
Switchgrass terpene synthase (TPS) gene expression in response to different treatments. **(A)** Transcript levels of selected TPS genes induced >10-fold in leaves (black bars) and/or roots (white bars). Samples were analyzed in biological and technical triplicate and normalized to the expression of the control gene elongation factor 1a (*ELF1*α). Control (C) expression levels were scaled to 1 for comparison of treatment effects. **(B)** Venn diagrams of the TPS genes presented in **(A)** and induced by different treatments. R, root; L, leaf; SA, salicylic acid; FAW, fall armyworm; MeJA, methyl jasmonate.

To determine whether correlations between transcript abundance and volatile terpene products could be established in response to treatment with FAW, MeJA, and SA, we selected multiple TPS genes for expression analysis by quantitative RT-PCR (**Figure 8A**). In leaves, substantial induction at the transcript level (>10-fold) following herbivory was observed for 12 TPS genes (*PvTPS01*, *PvTPS04*, *PvTPS05*, *PvTPS06*, *PvTPS08*, *PvTPS11*, *PvTPS14*, *PvTPS16*, *PvTPS19*, *PvTPS36*, *PvTPS53*, and *PvTPS56*), of which 10 genes and *PvTPS12* and *PvTPS15* were equally of more highly induced upon root treatment with MeJA (**Figure 8A**). SA-induced expression exceeding that in response to FAW and MeJA treatment was observed for *PvTPS04*, *PvTPS13*, *PvTPS16*, and *PvTPS53*. Highest induction of *TPS* transcript levels in roots was found for 11 genes in response to the application of MeJA (*PvTPS05*, *PvTPS06*, *PvTPS10*, *PvTPS11*, *PvTPS14*, *PvTPS17*, *PvTPS19*, *PvTPS20*, *PvTPS36*, *PvTPS53*, and *PvTPS56*) or both MeJA and SA (*PvTPS53*) (**Figure 8A**). For *PvTPS02*, *PvTPS03*, *PvTPS07*, and *PvTPS09*, induced transcript levels were lower than 10-fold in leaves and/or roots upon any of the treatments (**Supplementary Figure S5**).

For nine TPS genes, we were able to identify their enzymatic products as components of the induced volatile blends of leaves and/or roots: The genes encoding (*E*)-β-caryophyllene synthases (*PvTPS11*, *PvTPS14*, and *PvTPS19*) showed highest transcript abundance in leaves and roots upon treatment with MeJA (**Figure 8A**). (*E*)-β-Caryophyllene emissions from both tissues are most likely associated with the activity of these TPSs. Expression of *PvTPS16*, whose recombinant protein produced (*E*)-β-farnesene, was strongly induced by SA treatment in leaves and is likely be associated with (*E*)-β-farnesene emission from this tissue (**Figures 1** and **8A**). The gene encoding *Pv*TPS05, which was found to produce α-selinene volatiles, was most strongly expressed in roots by application of MeJA matching the detection of this compound from root tissue (**Figures 2** and **8A**). *Pv*TPS06 and *Pv*TPS09 both catalyze the formation of δ-cadinene, which was emitted from roots upon MeJA treatment (**Figures 2** and **5**). Only *PvTPS06* was highly induced by MeJA, indicating its likely function *in planta* (**Figure 8A**). Moreover, transcript levels of *PvTPS36* were substantially induced in leaves in response to MeJA application, although emission of one of the primary products of the *Pv*TPS36 enzyme, limonene, occurred only at low levels (**Figures 1** and **8A**). Interestingly, terpene products (cycloisosativene, borneol) associated with two genes (*PvTPS01* and *PvTPS04*), which showed highest expression in leaves upon FAW and/or phytohormone treatment, could only be detected in roots (**Figures 2** and **8A**, **Supplementary Figure S1**).

Some TPS genes with lower levels of induction may contribute to the emission of particular terpenes [e.g., the (*E*)-β-farnesene synthase gene *PvTPS02*]. Compounds produced *in vitro* by several other TPS enzymes could not be detected or occurred only at trace levels in leaves and roots despite a strong induction of their corresponding genes. For example, 1,8-cineole produced by *Pv*TPS08 was only detected in trace amounts in root tissues. Linalool, the single product of enzymes encoded by *PvTPS12*, *PvTPS13*, and *PvTPS15* (**Figure 6**), was neither detected in emissions from leaves and roots and may be further metabolized upon stress treatment. Other TPSs for which no associations could be established between their enzymatic products and volatile emissions include *Pv*TPS03 [(*E*)-γ-bisabolene synthase], *Pv*TPS10 (α-patchoulene synthase), *Pv*TPS17, and *Pv*TPS20 [(*E*)-β-bisabolene synthases], and TPS53 (geraniol synthase). Emission of germacrene D from roots may be associated with *PvTPS55*, the expression of which was not determined.

## Discussion

The switchgrass genome contains a large family of 44 predicted full-length mono- and sesqui-TPS genes, of which 32 genes encode functionally active proteins. Sesqui-TPSs belonging to the type-a subfamily make up the majority of this TPS group, while only few mono-TPSs have emerged in the type-a clade or are distributed over the type-b, type-g, and type-e/f clades (**Figure 3**). Similar distributions have been shown to occur in the TPS families of rice and sorghum (Chen et al., 2011). Expansions of type-a clades are also common in dicots, although typically a higher proportion of mono-TPSs can be found in the type-b and type-g clades of dicot species (Chen et al., 2011; Kulheim et al., 2015)

The size of the switchgrass mono- and sesqui-TPSs family is almost twice as large as the number of characterized or predicted proteins with mono- or sesqui-TPS activity in maize (Springer et al., 2018). Polyploidy likely contributed to the expansion of the switchgrass TPS gene family, which is in agreement with studies by Hofberger et al. (2015) demonstrating the role of polyploidy events in the diversification and expansion of terpene secondary metabolism. Gene duplication through polyploidization generates gene redundancy eventually increasing functional divergence and allowing species adaption (Wendel, 2000). As an allotetraploid, switchgrass evolved from two diploid ancestors giving rise to two complete subgenomes (N and K) and functional divergence of TPS genes. In *P. hallii*, a diploid relative of switchgrass, ∼32 putative full-length TPS genes are annotated (https://phytozome.jgi.doe.gov/), indicating that polyploidization of switchgrass more than doubled the number of TPS genes. Polyploidy events in domesticated grasses may not always result in large TPS gene families as has been suggested for wheat (Schmelz et al., 2014). However, in switchgrass, obligate outcrossing and limited breeding have maintained massive phenotypic and adaptive polymorphisms (Casler et al., 2007), in line with a higher level of diversification in TPS genes. Nevertheless, one-third of the TPS genes we characterized appear to be functionally inactive, while several other TPSs might have limited *in vivo* activity due to their subcellular localization, suggesting inactivation and loss of *in vivo* function for a substantial fraction of the gene family.

A comparison between the switchgrass subgenomes found that only 35 or 50% of the TPS genes on subgenome K and N, respectively, have syntenic orthologs on the other subgenome. This limited synteny indicates subgenome divergence in TPS gene organization. Syntenic regions include TPS genes with identical functions [*PvTPS14* and *PvTPS19*—(*E*)-β-caryophyllene synthases; *PvTPS17* and *PvTPS20*—(*E*)-β-bisabolene synthases], while other orthologs adopted different functional activities. Further comparison with the genome of the closely related diploid species *P. hallii* revealed syntenic orthologs for more than 15 switchgrass TPS genes on 6 of the 9 P. *hallii* chromosomes. Corresponding syntenic orthologs could also be identified for several of these genes on the genomes of the close relative *S. italica* and of *S. bicolor*. These findings are consistent with the observed collinearity between the switchgrass, *Setaria*, and sorghum genomes (Casler et al., 2011) and suggest the presence of ancestral TPS genes in the common progenitor of sorghum and switchgrass more than 20 million years ago. Syntenic regions on the sorghum genome include a cluster of TPS genes on chromosome 7, which was found to encode insect-induced sesquiterpene synthases and shares (*E*)-β-farnesene synthase activity (*Sorbic.007G055600*, *PvTPS109*) (Zhuang et al., 2012).

Most mono- and sesqui-TPS genes of switchgrass exhibit tissue-specific expression patterns (**Figure 7**). With the exception of the root-accumulated monoterpene borneol, the products associated with these TPSs could not be found in leaves and roots under constitutive conditions and became in part only detectable in response to stress treatment when gene expression was induced. It is possible that under nontreatment conditions enzyme activity or substrate levels are too low to result in detectable amounts of product. In roots, microbial activity may also metabolize terpene compounds as has been shown in vetiver grass (Del Giudice et al., 2008). It is also possible that the enzymatic products are further metabolized to nonvolatile derivatives. For example, β-macrocarpene, a volatile sesquiterpene olefin produced by two maize terpene synthases, is not detected in volatile blends because of its conversion to nonvolatile acid derivatives called zealexins, which function as pathogen-induced phytoalexins (Köllner et al., 2008b; Huffaker et al., 2011). In another study in maize, Ding et al. (2017) found that the volatile sesquiterpene β-selinene is a direct precursor of β-costic acid, a nonvolatile antibiotic acid derivative. Based on these findings, it is possible that α-selinene made by TPS05 in switchgrass roots serves as a precursor of α-costic acid that may exhibit similar functions in antimicrobial defense. Future analyses should be performed to identify possible oxygenated downstream derivatives of switchgrass TPS products.

Twelve TPS genes were found to be induced in switchgrass leaves upon feeding by FAW larvae. At least half of these genes are likely to contribute to the production of the volatile terpenes released upon FAW feeding based on the activity of their corresponding enzymes. The majority of the FAW-induced genes also responded to belowground treatment with MeJA, and two genes were induced by root treatment with SA indicating bottom–up systemic responses in *de novo* terpene biosynthesis (**Figure 8B**). While these effects are likely to be less pronounced with the application of lower concentrations of MeJA and SA or in response to actual root herbivory or pathogen infection, several studies have reported similar root-induced systemic responses in the metabolism of terpenoids and other secondary metabolites in photosynthetic tissues (Bezemer et al., 2003; Bezemer et al., 2004; Rasmann and Turlings, 2007; Erb et al., 2008; Kaplan et al., 2008). By contrast, much weaker systemic effects have been observed on root defensive metabolites including terpenes in maize upon shoot treatments or foliar feeding (Bezemer et al., 2003; Bezemer et al., 2004; Rasmann and Turlings, 2007; Erb et al., 2008; Kaplan et al., 2008). Our findings support this notion since FAW feeding did not cause a major increase in TPS gene expression in switchgrass roots and only a local treatment with MeJA could elicit such a response (**Figure 8B**).

The terpene olefins released by switchgrass leaves and roots upon insect or hormone treatment are frequently found in stress-induced volatile blends of other monocots and dicots (Unsicker et al., 2009; Massalha et al., 2017). While determining the function of these compounds is beyond the scope of this study, we assume that they play roles in direct and indirect defenses similar to those described previously in maize, rice, or other plants (Degenhardt et al., 2009; Hare and Sun, 2011; Taniguchi et al., 2014; Chen et al., 2018). A common constituent of herbivore-induced volatile blends in many plants including grasses is (*E*)-β-caryophyllene (Köllner et al., 2008a). This sesquiterpene, when released from damaged leaves of maize and rice plants, has been implicated in recruiting parasitoids of herbivores (Cheng et al., 2007; Köllner et al., 2008a; Yuan et al., 2008). We identified three (*E*)-β-caryophyllene synthase genes (*PvTPS11*, *PvTPS14*, and *PvTPS19*) (**Figure 5**), all of which are located on chromosome 2 and induced upon FAW feeding and treatment with MeJA. By contrast, in maize, rice, and sorghum, only single genes (*ZmTPS23*, Os08g04500, *SbTPS4*) have been associated with the synthesis of (*E*)-β-caryophyllene upon herbivore feeding (Köllner et al., 2008a; Zhuang et al., 2012; Chen et al., 2014). In MeJA-treated root tissue, *PvTPS14* was found to be induced approximately fourfold higher than *PvTPS11* and *PvTPS19* and may contribute to the emission of (*E*)-β-caryophyllene belowground. Induced root expression of (*E*)-β-caryophyllene synthases is common among grasses and has been implicated with recruitment of entomopathogenic nematodes for indirect defense against belowground herbivory (Rasmann et al., 2005).

(*E*)-β-Farnesene is another sesquiterpene that is released by many plant species and plays, among other volatiles, a role in indirect defense in maize (Schnee et al., 2006; Degenhardt, 2009). We found four TPS genes that encode functionally active (*E*)-β-farnesene synthases (**Figure 5**). However, only *PvTPS02* expression correlated with compound emission as a result of herbivore damage (**Figure 1** and **Supplementary Figure S5**). Another gene, *PvTPS16*, was highly expressed in leaves following SA treatment and strongly correlated with (*E*)-β-farnesene emission under this condition (**Figures 1** and **8A**). Despite limited and controversial evidence (Gibson and Pickett, 1983; Kunert et al., 2010), this response could potentially affect aphids, since (*E*)-β-farnesene serves as an alarm pheromone for many aphid taxa (Bowers et al., 1977; Pickett, 1983) and aphids are known to elicit both SA- and JA-dependent signaling pathways (Moran et al., 2002). A recent study by Donze-Reiner et al. (2017) found several TPS genes to be induced upon feeding by the grain aphid *S. graminum.* However, none of the (*E*)-β-farnesene synthase genes was among those induced by *S. graminum*, indicating that their expression might be suppressed. Instead, genes induced by aphid feeding included the (*E*)-β-bisabolene synthases *PvTPS17* and *PvTPS20* among other genes in the type-a family and genes in the type-c and type-e/f families, which have in part be characterized as diterpene synthases (Pelot et al., 2018). Whether these terpene compounds are produced upon *S. graminum* feeding is currently unknown.

We found only two monoterpenes (limonene and β-ocimene) to be emitted at low levels from treated switchgrass leaves (**Figure 1**). Except of *PvTPS36*, which was induced in leaves by MeJA treatment and makes limonene as an enzymatic product (**Figures 6** and **8A**), no terpene products of the other induced mono-TPS genes could be detected possibly because of the reasons addressed earlier. Interestingly, enzymatic products of two TPSs, the cycloisosativene synthase *Pv*TPS01 and the borneol synthase, *Pv*TPS04, could only be observed in emissions from roots, although the corresponding genes were most highly expressed in leaves upon FAW, MeJA, or SA treatment (**Figures 1** and **8A**, **Supplementary Figure S1**). Whether the absence of the compounds in leaf tissue is due to limited enzymatic activity, metabolization of the product, or transport from shoots to roots remains to be determined.

In summary, our study has provided a genetic road map for investigating the biosynthesis and function of volatile terpenoids in switchgrass. We have shown that the switchgrass genome contains an extended family of mono- and sesqui-TPS genes, several of which share syntenic orthologs in other grasses, exhibit tissue-specific expression, and respond to herbivory and phytohormone treatment above- and belowground. The volatiles associated with these genes and possibly their nonvolatile derivatives may exhibit functions in above- and belowground direct and indirect defense similar to those described for maize and other grasses. Further studies involving the generation of switchgrass mutants will evaluate these ecological roles in greater detail.

## Data Availability

The datasets generated for this study can be found in Phytozome, https://phytozome.jgi.doe.gov/pz/portal.html.

## Author Contributions

AM, XC, FC, and DT designed the study. AM, XC, TK, KP, and PZ performed bioinformatic analyses and gene annotation. JL performed synteny analyses. AM, XC, MR, LC, and SL performed enzyme characterizations. AM and XC performed RNA extraction, RT-qPCR, and stress treatments. AM and XC performed volatile profiling. AM, XC, FC, and DT wrote the manuscript. All authors reviewed, read, and approved the manuscript before submission.

## Funding

This work was supported by Community Science Program grant (WIP 2568) of the Department of Energy Joint Genome Institute and funds by the Translational Plant Sciences Program at Virginia Tech. The work conducted by the US Department of Energy Joint Genome Institute, a DOE Office of Science User Facility, is supported by the Office of Science of the US Department of Energy under contract no. DE-AC02-05CH11231.

## Conflict of Interest Statement

The authors declare that the research was conducted in the absence of any commercial or financial relationships that could be construed as a potential conflict of interest.
