# Supplementary material for: Biosynthesis and Emission of Stress-Induced Volatile Terpenes in Roots and Leaves of Switchgrass (Panicum virgatum L.)
Source: Front Plant Sci. 2019 Sep 19;10:1144. doi: 10.3389/fpls.2019.01144 (PMC6761604; doi:10.3389/fpls.2019.01144)
Supplement: Supplementary file 1 [file Table_1.pdf]

Supplementary Table 1. Normalized emission of volatile compounds from switchgrass plants (cv. Alamo) treated with fall armyworms (FAW), methyl jasmonate (MeJA), salicylic acid (SA) or mock control. Results from non-treated controls were the same as mock controls. Values were obtained in biological triplicate and are reported as mean peak area analyte/peak area internal standard (nonyl acetate)/gFW/hr ± SD. Significantly different emission rates among treatments were determined by ANOVA and post-hoc Tukey HSD tests where $\alpha \leq 0.05$. Significantly different values are marked with letters. Compound identification was based on similarity to library matches (NIST, WILEY) and comparisons to authentic standards. nd = not detected, tr = trace.

**Compound**	**Control**	**MeJA**	**FAW**	**SA**
limonene	nd	6.35e-04 ± 1.49e-04	nd	5.06e-04 ± 5.38e-05
(*E*)-β-ocimene	nd	2.31e-04 ± 8.76e-05[a]	8.63e-04 ± 1.62e-04[b]	nd
(*E*)-DMNT	nd	4.01e-04 ± 1.99e-04[a]	2.28e-03 ± 5.70e-04[b]	nd
indole	nd	5.71e-03 ± 6.16e-04	5.51e-03 ± 5.18e-04	nd
α-ylangene	nd	1.93e-04 ± 3.60e-05	nd	nd
elemene isomer	nd	2.26e-04 ± 7.82e-05	nd	nd
β-elemene	nd	5.03e-03 ± 6.89e-04[a]	1.74e-03 ± 3.00e-04[b]	nd
(*E*)-β-caryophyllene	nd	1.16e-02 ± 1.61e-03[a]	4.02e-03 ± 4.44e-04[b]	4.87e-05 ± 8.73e-06[c]
α-bergamotene	nd	4.90e-04 ± 1.53e-04[a]	1.49e-03 ± 3.12e-04[b]	7.04e-04 ± 4.54e-05[a]
unidentified sesquiterpene	nd	1.14e-04 ± 4.28e-05	5.32e-05 ± 6.75e-06	nd
(*E*)-β-farnesene	tr	2.47e-03 ± 9.97e-04[a]	6.12e-03 ± 3.55e-04[b]	6.14e-03 ± 3.77e-04[b]
α-humulene	nd	1.77e-03 ± 1.62e-04[a]	7.55e-04 ± 1.63e-04[b]	nd
β-copaene	nd	1.50e-03 ± 1.58e-04[a]	6.09e-04 ± 1.27e-04[b]	nd
**Total normalized emission**	**--**	**3.03e-02 ± 4.98e-03**	**2.34e-02 ± 2.96e-03**	**7.39e-03 ± 4.85e-04**
