# Supplementary material for: Biosynthesis and Emission of Stress-Induced Volatile Terpenes in Roots and Leaves of Switchgrass (Panicum virgatum L.)
Source: Front Plant Sci. 2019 Sep 19;10:1144. doi: 10.3389/fpls.2019.01144 (PMC6761604; doi:10.3389/fpls.2019.01144)
Supplement: Supplementary file 3 [file Table_3.pdf]

Supplementary Table 3. RT-qPCR primers used in this study.

	FWD	REV
*Pv*TPS01	GCCCCGCAGTCACAGTGC	CAGGTCGGAGGAGGCAAAGCTTTCC
*Pv*TPS02	GGATCACGGTAGAGGAGGCCAATG	CTTTGACACTGTATGGGTAGGTATACGC
*Pv*TPS03	GCACTTCATTCCAACAGTG	CGTTTATAATTTTCCTGACTTC
*Pv*TPS04	GGTTGAACCCTGATAATTCAGTGCCTAG	CAAGTGGATGGTATCCTTGAGGTTTTGAC
*Pv*TPS05	GCGGATGCGAGAGCGGG	CTGGCCCTGAATTGACGACGC
*Pv*TPS06	CTACTTCCGTTGCTACGACCATTCC	GGTTCATTGTCTCCACGACATTATTCTTGG
*Pv*TPS07	GTTTGGCTATCTTACCTGTTGG	GTCGGAGCTGCTGATG
*Pv*TPS08	GAGGTGGCTGGAGCAGCGG	CTCTCATGCTGGCCCCTAAAAGTGGAAC
*Pv*TPS09	CACCACCAAGGACTTTGTGGTTGTC	GCCATTCTTCGGACACATTAACCAAAGG
*Pv*TPS10	GCGATGGCGTACCAG	CCACGGATACACAGTC
*Pv*TPS11	GCCCTGGCCTCGCAACAAG	CCTGCTTGATCCGTGCACTCTCC
*Pv*TPS12	GTCACAGCCGGTTGGCCG	CATGGCTGAATTGGCACACCAGATC
*Pv*TPS13	CTGCACGCACCTTCTCCGTGC	CGGGCGCTGTTCCTACGTTC
*Pv*TPS14	GATCTCCGGCAGCCCTG	CAGCTTGCGAACCAC
*Pv*TPS15	CAGGGCCGTGAAGC	GACGGGACGCTCTG
*Pv*TPS16	CGCCTCCAGTTTGCTC	GCCGCTGTAGCGTG
*Pv*TPS17	GCCAGGAGGAGATCAAG	GGCAAAGTCGGCGG
*Pv*TPS19	GGACCTGCAGAGGCAG	CTCCGTGCCCTCTCC
*Pv*TPS20	GCGTCGGGGCATGAAG	GAATTTCCTGAAGTGAAGTTG
*Pv*TPS36	CTTCAAACCACCTCCTC	GGCGTTCTGACTGAG
*Pv*TPS53	CTATGTCACATGGAATGTGGC	GCCCTCTCTGTCTCTG
*Pv*TPS56	GAGCGAGGCTGCG	GCATCCGCTTCTCAG
ELF1a	GGAGAAGTCCCACATCAACATCGTGG	CGCTCCGCCTTGAGCTTGTC
