# Supplementary material for: Biosynthesis and Emission of Stress-Induced Volatile Terpenes in Roots and Leaves of Switchgrass (Panicum virgatum L.)
Source: Front Plant Sci. 2019 Sep 19;10:1144. doi: 10.3389/fpls.2019.01144 (PMC6761604; doi:10.3389/fpls.2019.01144)
Supplement: Supplementary file 4 [file DataSheet_1.pdf]

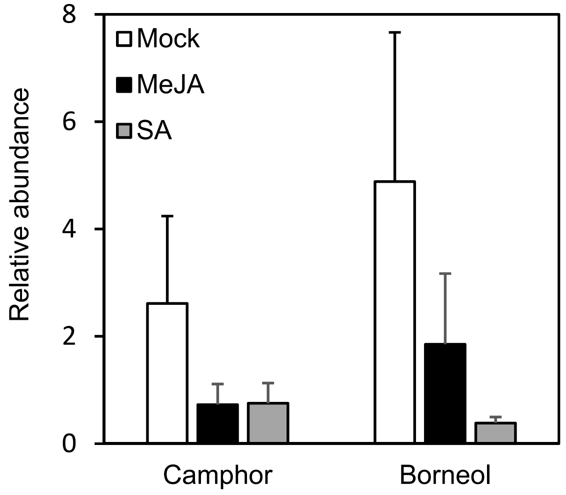

Supplementary Figure S1. SPME-GC-MS analysis of camphor and borneol emission following MeJA and SA treatment. Volatile compounds were analyzed in triplicate from detached pooled root material of 5-week old plants. Samples were normalized to an internal standard (1-bromodecane) and gram FW. Compound identification was based on matches with authentic standards (Sigma).

Supplementary Figure S2. Amino acid sequence alignment of switchgrass mono-TPSs and sesqui-TPSs. The multiple sequence alignment was generated using Clustal Omega (http://www.ebi.ac.uk/Tools/msa/clustalo/) with sequences from Supplementary Table S2 and visualized using BoxShade (http://www.ch.embnet.org/software/BOX_form.html1).

```
PvTPS15    1 MARAVKLLAAAGSHPLKACLGSGG------------------------------------
PvTPS106   1 ------------------------------------------------------------
PvTPS13    1 ------------------------------------------------------------
PvTPS02    1 ------------------------------------------------------------
PvTPS28    1 ------------------------------------------------------------
PvTPS27    1 ------------------------------------------------------------
PvTPS12    1 -------------------------------------------MSLKVTAGWPAA--AA-
PvTPS73    1 ----------------------------------------------MATPKWFQV--QRP
PvTPS52    1 -------MYTY--------------------IINAC----AVPGVAMATPKWFQV--RRP
PvTPS01    1 ------------------------------------------------------------
PvTPS85    1 ------------------------------------------------------------
PvTPS19    1 ------------------------------------------------------------
PvTPS14    1 ------------------------------------------------------------
PvTPS11    1 ------------------------------------------------------------
PvTPS26    1 --MQASVMTTLSLRPEGQCNGSFL---------------LASSTSRRPCISWSRRQQRPT
PvTPS04    1 -MEGS-VMTTFSLRP-GHCNGSFL---------------VAPATSRRPCISWRPRQQRQT
PvTPS83    1 -MQGTVTMTTLSLRPAGQCNGSCL---------------LAPATARQPRISWWRRQQRQT
PvTPS03    1 -ME-GSVMTTLSLRPAGQCNGSCL---------------LAPATARQPRISWWRRQQRQT
PvTPS62    1 MRGCTTLMATTSFKPQRN------------FHVAA----AATASTQCPSISWRQG--QKR
PvTPS08    1 MHSGSTVMTTFSSKPP-QC----SSRRSCHVHIGAAAASPPPLIRRCPSVSWRWLEQR--
PvTPS36    1 -------MTTFSFKPPPPCSS--SRRRSCNVHVGAAAASPPPLIRRCPSVSWRW--RLRR
PvTPS81    1 -------MTTFSFKPPPPCSSSSSSRRSCHVHIGAAAASPPPLIRRCPSVSWRWLQRLRR
PvTPS18    1 ------------------------------------------------------------
PvTPS16    1 ------------------------------------------------------------
PvTPS109   1 ------------------------------------------------MEVWNRDVHQQT
PvTPS69    1 ------------------------------------------------------------
PvTPS94    1 ------------------------------------------------------------
PvTPS17    1 ------------------------------------------------------------
PvTPS20    1 ------------------------------------------------------------
PvTPS10    1 ------------------------------------------------------------
PvTPS71    1 ------------------------------------------------------------
PvTPS79    1 ------------------------------------------------------------
PvTPS56    1 ------------------------------------------------------------
PvTPS05    1 ------------------------------------------------------------
PvTPS09    1 ------------------------------------------------------------
PvTPS50    1 ------------------------------------------------------------
PvTPS54    1 ------------MQPA-----------------------ASPEWIGMANVTWNVARGQVA
PvTPS33    1 --------------------------------------------------MAYVTRCRVP
PvTPS53    1 ----------------------------------------------MAYVTWNVARCQVP
PvTPS101   1 ------------------------------------------------------------
PvTPS07    1 ------------------------------------------------------------
PvTPS06    1 ------------------------------------------------------------
PvTPS55    1 ------------------------------------------------------------
PvTPS104   1 ------------------------------------------------------------
```

```
PvTPS15     25 ---------GQ--R---LGSLPRRTTT---------------------------------
PvTPS106     1 -----------------MAATPARTFSMPSVEPLLRSA-----------------SPAAT
PvTPS13      1 -----------------MAAAPARTFSVRSVEPLPVSA-----------------SLAAT
PvTPS02      1 -----------------MAAAPARTFSVRSVEPLPVSA-----------------SLAAT
PvTPS28      1 -----------------MAAAPAHVVFSTSVQLLLRSV-----------------SPAAA
PvTPS27      1 --------------------MAAHIFFVTSVRPLLLSA-----------------SPAAR
PvTPS12     15 ---TGRS--SR-------------------RHKLWC---------------------LSA
PvTPS73     13 CPPAGRSAVGK-------------------NTPARC------------------------
PvTPS52     28 CPAAR--PAGA-------------------NTPVRC------------------------
PvTPS01      1 ---------------------------------------------------M--ASPMIT
PvTPS85      1 -----------------------------------MG------------TYS--GSPVLA
PvTPS19      1 -----------------------------------MG------------TCR--GSPVLA
PvTPS14      1 -----------------------------------MG------------I-S--GSPVLA
PvTPS11      1 -----------------------------------MA------------T-C--GSHVLA
PvTPS26     44 K-----------------------------LHRQQMQ------------QRSVATVHHDN
PvTPS04     43 T-----------------------------LRRQQMQ------------QRSVVTD-HDN
PvTPS83     45 T-----------------------------LHRQQMQ------------QCSAATL-HND
PvTPS03     44 T-----------------------------LHRQQMQ------------QCSAATL-HND
PvTPS62     43 R-----------------------------HIRLQCH------------QLQH-------
PvTPS08     54 R-----------------------------HTRLQCG------------QSSPQ-QISNL
PvTPS36     50 H-----------------------------TCRLQCG------------QQSPQ-QNSNL
PvTPS81     54 H-----------------------------TCRLQCG------------QQSPQ-QNSNL
PvTPS18      1 ---------------------------------------------------------MSA
PvTPS16      1 ---------------------------------------------------MALTPPVCS
PvTPS109    13 Y-----------------------------KQQNQRSAREIIQVNKADGEKMALTPPVCS
PvTPS69      1 ---------------------------------------------------MALTPPVCS
PvTPS94      1 ---------------------------------------------------MAPASV---
PvTPS17      1 ---------------------------------------------------MASGHEDGP
PvTPS20      1 ---------------------------------------------------MASGHEDDS
PvTPS10      1 ------------------------------------------------------------
PvTPS71      1 --------------------------------------MVA--PREVGSSSMGLGSRV-I
PvTPS79      1 -----------------------------------MSSAIK--AT--SPVIQKIGSPME-
PvTPS56      1 -----------------------------------MAASEA--AS----------PPFS-
PvTPS05      1 -----------------------------------MAASEA--AS----------PPVS-
PvTPS09      1 ------------------------------------------------------------
PvTPS50      1 -----------------------------------------------------M-ENTNT
PvTPS54     26 ----IILGTGTQEHFYPR------------------------------------------
PvTPS33     11 HPENVIPGTRTHEHFHPHFLKTFYKY--RTMFPFHISRSNR--PG--NLVSMGM-ASSNA
PvTPS53     15 HPENNISGTGMQKHFHPHLAKPFFRY--RTTFPFHISRSNR--PG--NLVSMGM-ASCNA
PvTPS101     1 ----------------------------------------------------MA-SCRAR
PvTPS07      1 ---------MKSHNFHSRLANSRHPIISPLLLLYPGPSLVR--PT--RFSAVHI-KCTSA
PvTPS06      1 ------------------------------------------------------------
PvTPS55      1 -----------------------------------------------------M-APRTT
PvTPS104     1 ------------------------------------------------------------
```

```
PvTPS15    38 -------MPTTRRTRASRPGVNGVTQAH---KTFEV-EEHSNSPAK--------------
PvTPS106   27 R-------NGRPRGRSIIPW--AAAAS-----------NTLLPSN---------------
PvTPS13    27 R---NNDQRGRPRGRSIIAS--A-AAS-----------KTLLPSD---------------
PvTPS02    27 R---NNDQRGRPRGRSIIAS--A-AAS-----------KTLLPSD---------------
PvTPS28    27 G----G--IGRRGGR-IRPS----T-A-----------SNRPPSD---------------
PvTPS27    24 N----GRRGGRPGGC-IRTL----PAS-----------RTLPPSD---------------
PvTPS12    30 AES-PDHQQPRRRSANYRPSSWDYDALLSLK-----------GGGG--------------
PvTPS73    30 -SS-LPPPEPIRRSANYQPNSWSYASMESL------------AAAN--------------
PvTPS52    43 -SS-LPPPEPIRRSANYQPNSWSYASMESLL-----------EAAN--------------
PvTPS01     8 LAP-QSQ---CNQFREYSPSPWGDFFL-----------NHATCTPS--------------
PvTPS85    12 LAP-QDEDLLQRKPRPYTPSIWGDFFL-----------QHQPCTPS--------------
PvTPS19    12 LAS-HDE---ERKPRSYTPSIWGDFFL-----------KHQPCTPS--------------
PvTPS14    11 LAS-QDE---ERKPRPYTPSIWGDFFL-----------QHQPCTPS--------------
PvTPS11    11 LAS-QQEGLLQRKPRPYTPSIWGDFFL-----------KHQPCTPS--------------
PvTPS26    63 L-E-EVDDRLGKNPCNFHPSIWGDFFL-----------HYYDAAAA--------------
PvTPS04    61 L-E-ERKDGLGKNPCNFQPSIWGDFFL-----------HYYDTAAS--------------
PvTPS83    63 ----KVDDRLGKNPCNFHPSIWGDFFL-----------HYYDAAAS--------------
PvTPS03    62 ----KVDDRLGKNPCNFHPSIWGDFFL-----------HYYDAAAS--------------
PvTPS62    55 --D-DADNRLTRNTGIFHPSLWGDFFI-----------GYSNPAAP--------------
PvTPS08    72 --D-EDDSRLSQNAGIFHPSIWGDFFL-----------GYSNPAAA--------------
PvTPS36    68 V-D-EDDSRLSQNAGIFHPSIWGDFFL-----------GYSNPAAA--------------
PvTPS81    72 V-D-EDDSRLSQNAGIFHPSIWGDFFL-----------GYSNPAAA--------------
PvTPS18     4 L-H-SNSDEGLAKAPTFHPSLWGDFFL-----------TYQPPTAP--------------
PvTPS16    10 V-N-DVHQGQRKDRPTFHPSLWGDFFL-----------TYQPPTAP--------------
PvTPS109   44 V-N-DVHHGQRKDRPTFHPSLWGDFFL-----------TYQPPTAP--------------
PvTPS69    10 V-N-DVHHGQRKDRPTFHPSLWGDFFL-----------TYQPPTAP--------------
PvTPS94     7 D-H-DQKAEELRKATTFHPSLWGDFFL-----------TYQPPTAP--------------
PvTPS17    10 G-S-GQEEIKKPSASTFHPTLWGDFFL-----------SHKPPTLP--------------
PvTPS20    10 G-S-GQEQTKKPSASTFHPTLWGDFFL-----------SHEPPTSL--------------
PvTPS10     1 ---MEHVNPAKRPTVIVEDSKWTGFFI-----------ELQPLPC---------------
PvTPS71    20 AQG-YEVLDEERHAVDYRPSVWGDHFIKD---------PTLPHTN---------------
PvTPS79    21 ----T-IATTGAEDCPFEPSLWDDFF-V----------TYAPSIS---------------
PvTPS56    13 ----VEMMMGAAGLTGFEPCAWGDFFFI----------THAPPLS---------------
PvTPS05    13 ----VEMMMGAAGLTGFEPCVWGDFF-I----------TLAPPFS---------------
PvTPS09     1 ------------------------------------------------------------
PvTPS50     7 TKGFVVVNKLGRKTANYTPSRWSEFFI-----------DYVPPLV---------------
PvTPS54    40 -----------LRASDFHRTVWGNFFI-----------NHSPEPLQAIVHVL--EMLVLK
PvTPS33    64 NV------PE--RASDFHRTVWGDIFI-----------NYSPEPSQAWLTVYDSELLGLE
PvTPS53    68 NV------PETERASNFHRTVWGDFFI-----------NHSPEPLQ--------------
PvTPS101    8 RAK-EEEAGWKKMRGKRRGEEWDGPTASVLTGLPVDNSGGGGGRK---------------
PvTPS07    47 NKT-TASD----TVPVFHPSLWNDF--------FI---NYTPPPS---------------
PvTPS06     1 -MA-TSVATTI-PAPVFHPTVWGDYFI-----------NFTPEPL---------------
PvTPS55     7 SSD-EAATSWK-AAPEVHPSVWGDFFI-----------NYIPEPL---------------
PvTPS104    1 MAP-EAATSWK-AAPEVQPSVWGGFFI-----------NYIPEPL---------------
```

```
PvTPS15    73  -----------KFS--------------SHGISRQHNKGT--SSDTAMRKQLEQVDVLQN
PvTPS106   52  ------------FDSL----------------QHQILQQRR----ESGREMMATIDNLKR
PvTPS13    55  ------------FDLQLQE---------GLTSVQKILQERR----NSGREMMATIDNLKR
PvTPS02    55  ------------FDLQLQ--------------------ERR----NSAREIMATIDNLKR
PvTPS28    49  ------------LD--IQR---------SIMDVQEALRPCW----KRGREMVAAVDSLKR
PvTPS27    49  ------------HD--LQK---------SIMSVQKTLHGHP----KSGREVMAAVDNLKR
PvTPS12    64  -----------GRD---L--VCQFSHDQLKNSVKDMLLGKS----EESSCKATLIGTMQR
PvTPS73    62  -----------KYD---HNLADPSIFDKLMFRIRHLLLH-E----MELLPKLRVIDTLQR
PvTPS52    76  -----------KCD---HNQADPSIFDKLKFLVRHQLLH-E----MELLPKLRVIDTLQR
PvTPS01    39  -----------------QLLTMKERAQVKEENVRRIILESFASS--DLAQKLELVDTLQR
PvTPS85    46  -----------------QLLSMKESARIKQEEVRQIVLETTAS--RELVLKLELVDTLQR
PvTPS19    43  -----------------QLVSMKERARSKQEEVRQILLDTIAASS-ELVHKLELVDTLQR
PvTPS14    42  -----------------QLLSMEERAWSKQEEVRRILLDTIAASSELVVRKLELVDTLQR
PvTPS11    45  -----------------QLLSMKESARIKQEEVRQIILETTASC--ELVLKLELVDTLQR
PvTPS26    96  -------------S-SKQQTWMAERA-EVKEDVAKILASSVAW---DLDHRLQLIDALER
PvTPS04    94  -------------S-KQQTWMAAERADKVKQDVAKIVASSVAW---DLHHRLQLIDALER
PvTPS83    94  -------------S-KQQQTWMVEQADEVKQDVAKIVASSVAW---DLHHRLQLIDALER
PvTPS03    93  -------------S-K-QQTWMVEQADEVKQDVAKIVASSVAW---DLHHRLQLIDALER
PvTPS62    87  -----------------QQQRMEERADQLREEVAEIMAASSFS---SLHARLHMIDTLES
PvTPS08   104  -------------SSQ-QQTQMAERADKLKEQVAEMIASSTFR---GQHERLHLIDTLER
PvTPS36   101  -------------SSQQQQTQMAERADKLKEEVAGMIASSTVG---GQHGRLHLIDTLER
PvTPS81   105  -------------SSQ-QQTQMAELADRLKEEVAEMIACSTIG---GQHGRLHLIDTLER
PvTPS18    37  -----------------QHAYMKERAKVLKEEVRKIINGTN-----ELPKILDLILTLQR
PvTPS16    43  -----------------KRAYMTERAEVLKEEVRKMVKAAN-----EIPSILELIITLQR
PvTPS109   77  -----------------KRAYMTERAEVLKEEVRKMVKGAN-----EIPDILDLIITLQR
PvTPS69    43  -----------------KRAYMTERAEVLQEEVRKMVKGAN-----EIPDILDLIITLQR
PvTPS94    40  -----------------QHEYMKERAVVLREDVRKIVNGST-----DLRETMDLIITLQR
PvTPS17    43  -----------------KEIQMRERAAVLREEVRKIIKGSN-----DLPAMLDLIIMLQR
PvTPS20    43  -----------------QEIQMRERAEVLREEVRKIIKGSN-----DLPAMLELIITLQR
PvTPS10    32  -------------SHQQSEVRITDRRDELVQKVRCIIQDF-GE-KENLLQGMKTVDALER
PvTPS71    55  ---------------KKSLEWMEERRDALVSEAKKALTGTH-----DPMAEMKLIDAVQR
PvTPS79    50  ---------------QRSEKWMRERAAHLKKEVRHMFD--AHK-GLSVTNALILVDVLER
PvTPS56    44  ---------------QEAEKRMRERVEQLKGELRRRVFGAAGE-AMSVADTVALVDTLER
PvTPS05    43  ---------------QEAEKRMRERVEQLRGELRHRVFEAAGE-AMSVADTVALVDTLER
PvTPS09     1  ----------------------------MKEEIRAMITTAI-----DTVEIIKLIDTLEH
PvTPS50    41  ---------------NVSEEWLVEGAAKMKEEIRAMITTAI-----GTVEIMKLIDTLEH
PvTPS54    76  YGTICQIFVFPCFKRHKSEEWMTEKANQLKKKISELFEACT-----TPVEQLELVDTLQH
PvTPS33   105  YGTIC----------HKSEEWMTKKANQLKRRISELFETCT-----TPVEQLELVDTLQH
PvTPS53    97  ----------------KSEEWMTEKANQLKKKISELFEACT-----TPVEQLELVDTLQH
PvTPS101   52  ---------------ERSEKWMRERANKLKEEVGVLFQNCK-----NIVEKMNLVDVLQR
PvTPS07    76  ---------------QRSEKWMRERANKLKEEVGVLFQNCK-----NIVEKMNLVDVLQR
PvTPS06    32  ---------------QISDEKMAERINQLKEDVSGMFQAAKN----NVVETMNLVDVVQR
PvTPS55    39  ---------------QVSEEKMIERADKLKGEVCGLFEACK-----NVVEKLDLVDVLQR
PvTPS104   33  ---------------QVSDEKMIERVNKLKGEVCGLFEACK-----NVVEKLDLVDVLQR
```

```
PvTPS15    106 MGISRHFDGEIKRILDMTYSCWLQ------GD-EDIILDAGTCAMAFRILRMNGYDVSA-
PvTPS106    80 LCIDHYFEEEIQSAMGACMHL-------------IHSDDLFDATLAFRLMREAGHDVSAD
PvTPS13     90 LCIDHYFQEEIQSAMGACLDL-------------IHSDDLFDATLAFKLVREAGHDVSAD
PvTPS02     79 LCIDHYFQEEIQSAMGACLDL-------------IHSDDLFDATLAFKLVREAGHDVSAD
PvTPS28     82 LCIDHYFQEEVESAMAVAKDL-------------VRSDD-LDATLSFRLMREAGHDVSSD
PvTPS27     82 LCIDHYFEEEVESAMAVAVDL-------------VRSDDLLDATLSFRLLRETGHDVSAD
PvTPS12    104 LGISYHFEEDIRNILSSISMEIAN---------DRRVDDVASIALKFRLLRENGFSADPG
PvTPS73    103 LGVAYHFDEEISDLLKSVSMEGQD---------IDRMDDAHLMTLLFRLLRQSKSPTSPE
PvTPS52    117 LGVAYHFDEEIRAVLNSVTMEGQD---------VDRMDDAHLMTLLFRLLRQNKSPTSPE
PvTPS01     80 IGVDYHYKKEINDLLYSIYN-----------DEDGGSDDLYVTSLRFYLLRKHGYTVSA-
PvTPS85     87 IGV------EIDELLRDVHLDAQH-------EE-GCDDDLYVVSVRFYLLRKHGYNVSS-
PvTPS19     85 IGVDYHYKKEIDELLRDVVHDPQH-------EEGCDDDELYVVSLRFYLLRKHGYNVSS-
PvTPS14     85 IGVDYHFKKEIDELLRDIHGSQ-R--------EGGHDDELYLVSLRFYLLRKHGYNVSS-
PvTPS11     86 IGVNYHYRKEIDELLRDIHGSK-H-------EEEGCDDELYLVSLRFYLLRKHGYNVSS-
PvTPS26    138 LCLDHLFEDDINAALTQIRTS------------NVTDCY--------------GYRVSP-
PvTPS04    137 VCLDYLFEDDINATLTEIRTA------------NLTDCDLHTVAMWFYQLRKHGHRVSP-
PvTPS83    137 LCLDHLFEDDINAALTQIRTV------------NLTDCDLHTVAMWFYLLRKHGHRVSP-
PvTPS03    135 LCLDHLFEDDINAALTQIRTV------------NLTDCDLHTVAMWFYLLRKHGHRVSP-
PvTPS62    127 LCLDHLFEQEINDALQQVVAA------------DVRDCDLGTVALWFCLLRKHRYMVSP-
PvTPS08    147 LCLDHLFEEDISAALSQIEAA------------GVSDCDIGTVALWFCLLRKHRYRVSP-
PvTPS36    145 LCLDHLFEEEISAALPQIEAA------------GVSDCDLGTVALWFSLLRRHRYRVSP-
PvTPS81    148 LCLDHLFEEEISAALPQIEAA------------GVSDCDLGTIALWFSLLRKHRYRVSP-
PvTPS18     75 -------------HLRFVYNS------------GYDVNDLNLVSLRFYLLRKNGYDVPS-
PvTPS16     81 LGLDNYYENEIDEQLLFVYDS------------EYDDKDLNLVSLRFYLLRKNGYDVPS-
PvTPS109   115 LGLDNYYENEIDDKLLFVYDS------------EYDDKDLNLVSLRFYLLRKNGYDVPS-
PvTPS69     81 LGLDNYYENEIDDKLLFVYDS------------EYDDKDLNLVSLRFYLLRKNGYDVPS-
PvTPS94     78 LGLDYYYENEIDKLLQDIHNL------------DYNDKDLNLVSLRFYLLRKNNYDVSS-
PvTPS17     81 LSLDYHYEDEINKLLHIVYNS------------NHYDGDLNIVSRRFYLLRKSGYNVPS-
PvTPS20     81 LSLDYHYEDEINEMLLIVYNS------------NHYDGDLNVVSHRFYLLRKSGYYVPS-
PvTPS10     77 LGVGYHFEQEIATFMDVLSSRKP-----------AVGDDLCAVALQFRLLRQHHYDATC-
PvTPS71     95 LGISYHFQDEIHASLQKLK--SIE----------FDSESLYQISLQFWLLRQERYYVSC-
PvTPS79     92 LGIDNHFEEEICAVLCRVHSEEQE---------FDNSKELHIVALRFRLLRQHGFFVST-
PvTPS56     88 LGVDAHFREEIGAALRRVVVVNGESCSDNSGSGSCPDSDLRVVALRFRLLRQHGYWVPT-
PvTPS05     87 LGVDSHFREEIGAALRRVV---------NSGPGSCKDGDLRVVALRFRLLRQHGYWVPT-
PvTPS09     28 LCLDYHFEAEINDKLQFLN--GTK----------FDSDDLHQVALRFRRLRQRGFYVPP-
PvTPS50     81 LCLDYHFEAEINDKVQFLN--GTK----------FDSDDLHQVALRFRLLRQRGFYVPP-
PvTPS54    131 LSIDHHFKKQILDILSSIH--DTE----------FNSTCLHEVALRFRLLRQQGFWVSPV
PvTPS33    150 LSIDHHFKKQILAILSSIH--DTE----------FNSTCLHEVALRFRLLRQQGLWVSP-
PvTPS53    136 LSIDHHFKKQIHDILSSIH--DTE----------FNSTCLHEVALRFRLLRQQGFWVSP-
PvTPS101    92 LGIDHHFEEQITTTLHSIH--NAD----------FNSGSLNEVSLRFRLLRQQGFWVPP-
PvTPS07    116 LGIDHHFEEQITTTLHSIH--NAD----------FNSGSLNEVSLRFRLLRQQGFWVPP-
PvTPS06     73 LGIDHHFEEQIATALARLH--SAD----------FNSSSLHEVALRFRLLRQQGFWVSP-
PvTPS55     79 LGIDHHFQEQIATTLSIIHNQRDE----------FNSSDLHEVSLRFRLLRQHGFWVPA-
PvTPS104    73 LGIDHHFQEQIATTLSSIH--RDE----------FNSSNLHEVALRFRLLRQHGFWVPA-
```

```
PvTPS15   158 DELHHVAEALGFHPSLEGYLNDTRSLLELHKASKVSISEDESILDFIGSWSGCLLKEQLR
PvTPS106  127 DVLRRFTDGNGE--FTLALSKDVRGLLSLHDMSHLDMGVET-SLHKAKEFSSKHLAS--A
PvTPS13   137 DVLRRFTDGTGE--FKLALSKDIRGLLSLHDMSHLDMGEEP-SLHKAKEFSSKHLAS--A
PvTPS02   126 DVLRRFTDDTGE--FKLALSKDIRGLLSLHDMSHLDMGEEP-SLHKAKEFSSKHLAS--A
PvTPS28   128 DLLRRFTDAAGE--LSL-LTKDIWALLSLHDMSHLDMGEEA-SLHKANDFSSKHLAC--A
PvTPS27   129 DLLRRFTDGAGE--LSLALTEDIWALLSLHDMSHLDMGEEASSLHKAKDFSSKHLAS--A
PvTPS12   155 LLLKHDTYAKQC--SKGTLQRDVNRFLSINEASYLAFRG-EEMLDLARKFSTKALKD--L
PvTPS73   154 LLLRSLKDATGG--FKKSLREDTQGLLSLYEASHLAFGG-EDILDEARVFSTEALRE--R
PvTPS52   168 LLLRRLKDGKGG--FKKSLREDTQGLLSLYEASHLAFGG-EDILDEARVFSTEALRE--R
PvTPS01   128 DVFEKFRDKQGN--IS---SDDVSCLLMLYDAAHVRTHG-EEILDDMITFNKSRLQS--L
PvTPS85   132 DEFVKFRDDQGN--FAT--SNDVKCLLALYDAAHLRTRG-EEILDNAIAFTKSRLQS--M
PvTPS19   137 DVFVKFKDDCGN--FA---SNDVKCLLALYDAANLRTRG-EEILNSAVVFIRSRLQS--M
PvTPS14   135 DVFAKFKDDHGN--FA---SNDAKCLLVLYEAAHLRTRG-EEILDNAVVFTRSRLRS--M
PvTPS11   137 DVFVRFRDNQGN--FT---SNDVKCLLALYDAAHLRTCG-EEILDNAMAFTKSHLQS--M
PvTPS26   171 DVFVRFKDQEGS--FL---ANNPVELLSLYNAAHLGTHG-EIILDEAAIFTRTRLET--I
PvTPS04   184 DVFMRFKDQEGS--FL---AKSLVELLSLYNAAHLGTHG-EIILDEAIVFTRSRLET--M
PvTPS83   184 DVFVRFKDQEGS--FL---ANNPVELLSLYNAAHLGTHG-EIILDEAVVFTRTHLEA--I
PvTPS03   182 DVFVRFKDQEGS--FL---ANNPVELLSLYNAAHLGTHG-EIILDEAVVFTRTHLEA--I
PvTPS62   174 EVFLRFKDEKGG--FL---SDNPIDLLNLYNAAHMRTLG-EILLDEAILFTRRRLEA--V
PvTPS08   194 DVFARFKNEEGG--FL---ADNPEDLLSLYNAAYMGVHG-ETILDEAILFTRRRLET--A
PvTPS36   192 DVFARFKNEEGG--FL---ADNPEDLLSLYNAAHMGTHG-ETILDEAIVFTRRRLET--T
PvTPS81   195 DVFARFKNEEGG--FL---ADNPEDLLSLYNAAHMGTHG-ETILDEAIVFTRRRLET--T
PvTPS18   109 DVFLNFKGMEGR--FA---FDDIRGLLSLYNAAYLRTHG-EKVLDEAIIFTRSHLEA--M
PvTPS16   128 DVFKSFKDNEGN--FV---ADNTKTLLNLYNAAFLRTHE-DKILDEALLFTRSQLEA--V
PvTPS109  162 DVFKSFKDNEGK--FV---ADNTKTLLSLYNAAHLRTHG-DKVLDEAIIFTKSQLEA--V
PvTPS69   128 DVFKSFKDNKGK--FV---ADNTKTLLSLYNAAHLRTHG-DKVLDEAIIFTKSQLEA--V
PvTPS94   125 DVFLNFKIQDGN--FA---IADTRSLLSLYNAAYLRRHG-DKVLDEAISFTRCCLQD--I
PvTPS17   128 DVFLNFKDKQGN--FV---DADIRSLLSLYNAAYLRTHG-EPLLDEAISFTRRCLQG--G
PvTPS20   128 DVFLNFKDKQGN--FV---DADIRSLLSLYNAAYLRTHG-EPLLDEAISFTRRCLQG--G
PvTPS10   125 DVFKTFLDDNGD--FKDALRSDVDALLSLYEAAHVSKCN-EDVLNRAVVFTVDRLSYLAN
PvTPS71   142 DVFQSFMDNQQN--LKVSLASDVRALLALYEAAHLGTPD-EQLLTEAQRQTTSLLKS--M
PvTPS79   142 DVFNKFKDGTGN--FSTGLTGDPRGLLSLYNGAHMAVPG-EDVLDDAITFTRSHLKA--M
PvTPS56   147 DVFDRFRDATGS--FRASLSSDPGSLLSLYNAAHMAIPG-ERVLYEAISFCRRHLES--M
PvTPS05   137 DVFDRFRDDTGS--FRASLSSDPGGLLSLYNAAHMATPG-ERVLDEAISFCRRHLES--M
PvTPS09    75 DVFNKFKDEFGN--FKTTLIGDTKGLLSLYNATHLLLPN-ETLLEDAIKFAGDNLKK--A
PvTPS50   128 DVFNKFKDEFGN--FKTTLIGDTKGLLSLYNAAHLLLPN-ETVLEDAIKFAGDNLKK--A
PvTPS54   179 DEFNRFKDENGN--FIVEITNDARGLL---------------------------------
PvTPS33   197 DEFNRFKDENGN--FNVEITNDARGLLSLYNAAYLLTHG-EPELEEAILFARQHLES--M
PvTPS53   183 DEFYRFKDENGN--FIVEITNDARGLLSLYNAAYLFTHG-EPELEEAILFARQHLES--M
PvTPS101  139 DVFNIFKSGDGS--FVSDITNDTKGLLGLYNAAHLLTHN-EGALEEAILFARHHLEL--E
PvTPS07   163 DVFNIFKSGDGS--FVSDITNDTKGLLGLYNAAHLLTHN-EGALEEAILFARHHLEL--E
PvTPS06   120 DEFNKFKNEDGS--FISGIENDPKGLLSLYNAAHLLTHD-EGTLEDAILFSRRHLES--I
PvTPS55   128 DEFDRFKLEDGS--FIDSIANDPKGLLSLYNAANLLTHNNEGALEEALMFARRHLEL--I
PvTPS104  120 DEFDKFKLEDGS--FIYSIANDPKGLFSLYNAANLLTHN-EGALEEALIFARKHLEL--I
[illegible]
```

```
PvTPS15   218 SGGLQG-TPLFREVEHALEFPFYTTLDRLD-HRWNIENFNITGEKMLKTSSMLCSTNDDI
PvTPS106  182 IRHLEP--GLARYVRQSLDHPYHLNLMQYK-ARHHLSYLQS---------LPTRN--IAM
PvTPS13   192 IRYLEP--GLARYVRQSLDHPYHLSLMQYK-ARHHLSYLQS---------LPTRD--TAM
PvTPS02   181 IRYLEP--GLARYVRQSLDHPYHLSLMQYK-ARHHLSYLQS---------LPTRD--TAM
PvTPS28   182 IRYLNP--DLARYVRQSLDHPYHLILMQYK-ARHHLSYLQS---------QPNRKSTAAI
PvTPS27   185 IRYLNP--GLARYVRQSLDHPYHMSLMQYK-ARHHLCYMQS---------LPNRKSTAAM
PvTPS12   210 MPSMPP--HTRKRVAHALDLPLHWMAPRLE-TRWFIDHCAGD---------IGLH--PLL
PvTPS73   209 MPLMRP--HLRSSVNNALAVPLHWAAPRLQATRWFIDQK---------------------
PvTPS52   223 MPLMRP--HLRSSVDNALAVPLHWAAPRLQA-RWFIGHYAGD---------GGAD--QVM
PvTPS01   180 TMKNLE-PELAEEVRCTLETPRFRRVERVE-ARLYISVYEK---------KAVHD--GTI
PvTPS85   185 IETL-D-PELAAEVEYTLETPSYRRVERVE-ARRYISVYEK---------RATRN--DTI
PvTPS19   189 MKTLDD-PELAAEVEYTLETPSYRRVERVE-ARRYISLYEK---------KVTRN--DTI
PvTPS14   187 VKTL-D-PELAAEVEYTLETPSYRRVERVE-ARRYISLYEK---------KVTRN--DTI
PvTPS11   189 IKTL-D-PELAAEVEYTLETPSYRRVQRVE-ARRYISLYEK---------KVRRN--DAI
PvTPS26   223 LPSLEG--SLAHEIKCALEIPLPRRVRIFE-SKYYVSRFEN---------EITVH--DSV
PvTPS04   236 LPSLEG--SLAHEIKCALEIPLPRRVRIYE-SKYYISRFEK---------EVTVH--KSI
PvTPS83   236 LPSLEG--SLAHEIKCALEIPLPRRVRIYE-SKYYVSTFEK---------DVTVH--DTV
PvTPS03   234 LPSLEG--SLAHEIKCALEIPLPRRVRIYE-SKYYVSTFEK---------DVTVH--DTV
PvTPS62   226 LPYLEG--SLAREVNSALETPHPRRLRIYE-SKYYISAYEK---------DGTVH--EKV
PvTPS08   246 LSNMEE-SLLAHEIKSALEIPSPRRVRIYE-SKHYISAYEK---------DATVH--ETV
PvTPS36   244 LRDMEEESSLLAREIKSALEIPLPRRVRIYE-SKHYISAYEQD--------DATVH--ETV
PvTPS81   247 LRDMEEESSLLAREIKSALEIPLPRRVRIYE-SKHYISAYEQD--------DATVH--ETV
PvTPS18   161 -------------KSQSLQTPLFRRVRILE-TRNYIPIYEM---------EPSQN--MAI
PvTPS16   180 LDSLES--TLADEVSHALQTPLFRRIRILE-TRTYIPIYEK---------EAARN--EVI
PvTPS109  214 LDSLES--TLADEVSHALQTPLFRRIRILE-TRTYIPIYEK---------EAARN--EVI
PvTPS69   180 LDSLES--TLADEVSHALQTPLFRRIRILE-TRTYIPIYEK---------EAARN--EVI
PvTPS94   177 VEHSES--PFAKEVSSSLHTPLFRRVGILE-ARNYIPIYEK---------EATRN--EAI
PvTPS17   180 LENLES--PLAEEVSCALDTPLFRRVGILE-TRNYIPIYEK---------EATQK--EAI
PvTPS20   180 LENLES--PLAEEVSCALDTPLFRRVGILE-TRNYIPIYEK---------EATRK--EAI
PvTPS10   182 GGSLPK--PIQNKVLHALAAPTYRRMKRLQ-AKLYISIYDDD---------NEKD--HDI
PvTPS71   197 GDHLEK--PLADKVRHALQTPSFRRMKRLE-ARQYIPLYEQD--------KEDCN--ELA
PvTPS79   197 KGNLGY--PIAGQVARALDIALPRYMPQLE-TIHYIIEYEQE---------DVHN--ATI
PvTPS56   202 RGELAS--PMAEQVSRALDIPLPRLPKRLE-TVRYIAEYARE---------EGHD--PVA
PvTPS05   192 RGELAS--PLAEQVSRALDIPLPRLPKRLE-TVRYIVEYEKE---------EGHD--PVA
PvTPS09   130 VHGLKS--PLAEQVPRALKSPLPRFMQKLE-ARFYIDEYGDE---------EDSN--DTI
PvTPS50   183 VHDLKS--PLAEQVSRALKSPLPRFMQKLE-ARFYIDEYGDE---------EDSN--DTI
PvTPS54   204 -------------MNRAFHLPLSRTLRRLE-ALHYISEYKGE---------PTPN--SSI
PvTPS33   252 RNTLN----------------------------------TRE---------PTHN--SSI
PvTPS53   238 RNKLEY--PLAQQVNRALHLPLSRTLRRVE-ALNYISEYKGE---------PTHN--SSI
PvTPS101  194 RSTLKS--PFAEQVTRALRIPLPRTLKRVE-ALNYITEYNVY--E-----QPYNP--A-I
PvTPS07   218 RSTLKS--PFAEQVTRALRIPLPRTLKRVE-ALNYITEYNVY--E-----QPYNP--A-I
PvTPS06   175 QSSLKP--PLADQVGRALQIPLPRTLKREE-AISFIPEYSSSVQD-----QTYSP--EIL
PvTPS55   184 QSSLKS--PLADQVARALKIPLPRTLKRVE-AVSYMQEYSVE--------QRYNP--A-I
PvTPS104  175 QSSLES--PLADQVARALKIPLPRTLKRVE-AVSYMQEYSVD--------Q---------
```

```
PvTPS15   276 LALGIRDFSASQVTYQEELRHLKSWVKESRLD-QL-PFARQKLEYFYLSAAGTIFTPELS
PvTPS106  228 EELAIAEFQLNKLQHQQEMQEIKRWWMDLGLAQEI-PVARDQVLKWYMWPMSILQGSSLS
PvTPS13   238 EGLAIAEFQLSKQLHQEEMQEVKRWWMDLGLSHEI-PVVRDQVPKWYVWAMTSLQGPSLS
PvTPS02   227 EGLAIAEFQLSKQLHQEEMQEVKRWWMDLGLSHEI-PVVRDQVPKWYVWAMTSLQGPSLS
PvTPS28   230 EELATAEFHLNKLLHQKEMEEVKR------------------------------------
PvTPS27   233 EELATAEFHFNKLLHQKEMEEVKRWWMGLGLAQEV-PVARDQVLKWYMWSMTIIQGSSFS
PvTPS12   256 LQFAKVDFDNVQRAHQEELARLTKWWRDIGLCDKL-TFSRDRLMECFHYANGIVWEPKHG
PvTPS73   246 -----------------E---LSRITRNADLNKKL-PFARDRLMECFYFATGVASEPSLA
PvTPS52   269 LRFAKIDFNNVKKLHQQELARITRWWRNAGLNKKL-PFARDRLMECFYFATGVASEPSLA
PvTPS01   227 LEFAKLDYNILQAIYCDELKELTIWWKDLHSKTDL-SFARDRMVEIHFWILGTIYEPYYS
PvTPS85   231 LEFAKLDYNILQALYCEELKALTLWWKGLQSQAYA-RFARDRVAEMHFWMLGIVYEPHQS
PvTPS19   236 LEFAKLDYNILQALYCEELKVLTAWWKGLQSQACV-RFARDRVPEMHFWMLGIIQEPRQS
PvTPS14   233 LEFAKLDYNILQALYCEELKALTAWWKALQSQAYA-RFARDRVTEMHFWMLGVIHEPCQS
PvTPS11   235 LEFAKLDYNILQALYCEELKALTVWWKGLQSQAYA-RFARDRVTEMHFWMLGVIHEPRQS
PvTPS26   269 LQLAKLNANITQLHHQQELEIVTRWWRGLEIESKL-PFARDRVVESYFWMLGVYFEPCHS
PvTPS04   282 LQLAKLNSNIMQLHHQQELEIITRWWKDMDIESKL-PFARDRVIECYFWILGVYFEPCHS
PvTPS83   282 LQLAKLNSNIMQLRHQQELEIITRWWKGLEIESKL-PFARDRVVECYFWIVGVYFEPCQS
PvTPS03   280 LQLAKLNSNIMQLRHQQELEIITRWWKGLEIESKL-PFARDRVVECYFWIVGVYFEPCQS
PvTPS62   272 LQLAKLNSNIMQLHHQHELAIISRWWKDVQIESRL-PFARDRVVECYLWILGVYFEPCYS
PvTPS08   293 LQLAKLNSNIMQLHHQHDLEIITRWWKGLHVESKL-PFARDRVVECYLWILGVYYEPCYS
PvTPS36   293 LQLAKLNSNIMQLHHQHDLEIITRWWEGLHVESRF-PFARDRVVECYLWILGVYYEPCFS
PvTPS81   296 LQLAKLNSNIMQLHHQHDLEIITRWWEGLHVESRF-PFARDRVVECYLWILGVYYEPCFS
PvTPS18   196 LEFAKLNFNLLQLLYCE-LKMVTLWWKQLNVETNL-SFIRDRIVETHFWMAGACSEPKYS
PvTPS16   226 LEFAKLNFNLLQLLYCEE------WWKQLNVETNL-SFIRDRIVEMHFWMTGACSEPQYS
PvTPS109  260 LEFAKLNFNLLQLLYCEELKTVTLWWKQLNVETNL-CFIRDRIVEMHFWMTGACSEPQYS
PvTPS69   226 LEFAKLNFNLLQLLYCEELKTVTLWWKQLNVETNL-CFIRDRIVEMHFWMTGACSEPQYS
PvTPS94   223 LEYAKLNFYLQQLVFCEELKHCTMWWKEFLVKSKM-TFVRDRIVEVYFWMNGACYDPPYS
PvTPS17   226 LEFAKLNFNLLQLLYCEELKDVTAWWKNLNVEANF-HFVRNRIVEMYFWMNGACHEPQYS
PvTPS20   226 LEFAKLNFNLLQLLYCEELKDVTAWWKNLNVEANF-YFVRNRIVEMYFWMNGACHEPQYS
PvTPS10   228 LELAKLDFHILQQMHRDEVRSICLWYKDLNPKSTIGQYIRERPVECYYWALGAFYEPHYA
PvTPS71   244 LELAKLDFYLLQRIHREEVKEICEWYHGLESPQRL-FYARHRPTEAYFWALGVYYEPQYA
PvTPS79   243 LELARLDYNLRRSAHLKELRTFCSWWKDIYEDVKL-PYSRDRSVEMYFWAFGAFQREHNS
PvTPS56   248 LELARLDFDLVRALHLEELRALSLWWKEVYGDVKL-SYARDRLVENYFWTCGVFHEEEYS
PvTPS05   238 LELARLDFDLVRALHLEELRALSLWWKEVYGDVKL-SYARDRLVENYFWTCGVFHEEEYS
PvTPS09   176 LKFAKLDFIAVQREHCKELKALSLWWKDLRITETL-PYARDRIVECYFWILGVYFEPCYS
PvTPS50   229 LKFAKLDFIAVQREHCKELKALSLWWKDLRITETL-PYARDRIVECYFWILGVYFEPCYS
PvTPS54   239 LEFAKLDFVLLQRLHLKELKALSRWWKDLYNEEGL-AYSRDRVVECYLWSYTAYYETEYS
PvTPS33   267 LEFAKLDFVLLQRLHLKELKALSRWWKNLYDEDGL-AYSRDRVVECYLWSYTAYYEKEYS
PvTPS53   284 LEFAKLDFVLLQRLHLKELKALSRWWKDLYNEEGL-AYSRDRVVECYLWSYTAYYEKEYS
PvTPS101  241 LELAKLEFNLLQHLYLKELKTVSQWWKDLSAYIEL-DYIRDRLIEGYFYSYNVYHEQEHA
PvTPS07   265 LELAKLEFNLLQHLYLKELKTVSQWWKDLSAYIEL-DYIRDRLIEGYFYSYNVYHEQEHA
PvTPS06   225 LELAKLDFNLLQHLYQKELKALTQWWKDLSGEIGL-DYVRDRIVECYFWSYSVHYEQANA
PvTPS55   230 LELAKLDFNLLQRLHQKELKTISQWWKDLSEDVGL-EYVRDRIVECYFWAYSMYYEQEYA
PvTPS104  215 ----------------------RWWKDLSEDIGL-EYMRDRIVECYFWAYSMYYEQEYA
[illegible]
```

```
PvTPS15    334 DARILWAKNGVLTTIVDDFFDVGGSKEELENLVTLAE-----------------------
PvTPS106   287 RNRIEITKIISLVYVVDDIFDLVGTLEELSLLTKAIK-----------------------
PvTPS13    297 GYRIDTTKIISLVYVVDDIFDLVGTPEELSLFTQAIK-----------------------
PvTPS02    286 GYRIDTTKIISLVYVVDDIFDLVGTPEELSLFTQAIK-----------------------
PvTPS28    254 -YRVQLAKIISLIYIVDDLFDLVGTQEELSLFTKAVK-----------------------
PvTPS27    292 RFRVQLTKIISLIYVVDDLFDLVGTQEELSLFTKAVK-----------------------
PvTPS12    315 ACREMLARVANLIIHLDDVYDVYGTLDELILFTDAIG-----------------------
PvTPS73    285 ACREVVAKAFALIAVLDDIYDIYGTLDELAVFTDAIG-----------------------
PvTPS52    328 ASREVVAKAFALIVVLDDIYDIYGTLDELAVFTDAIE-----------------------
PvTPS01    286 YSRIVVTKFTLLASLLDDLYDNYCCTTEESTIFNT-------------------------
PvTPS85    290 YARMALAKWLKLVSLMDDICDNYSTTEEYD-CSFLLW-----------------------
PvTPS19    295 YARIALTKCFKLVSLMDDLCDNYSTTEEYE-IFMT-------------------------
PvTPS14    292 YARIALTKCFKLVSLMDDFCDNYSDTEEFE-IFIT-------------------------
PvTPS11    294 YARIALTKCFKLVSLMDDFCDNYSDTEEFE-IFIT-------------------------
PvTPS26    328 RGRIILTMIIAIIGLLDDMFDSYGTMEECELLTNCIEKVSI-------------------
PvTPS04    341 RGRIILTMVIAIATIFDDTFDSYGTMEECQLLTNCME-----------------------
PvTPS83    341 RGRIILTMVIAIIGIFDDIFDSYGTMEECELLTNCVE-----------------------
PvTPS03    339 RGRIILTMVIAIIGIFDDIFDSYGTMEECELLTNCVE-----------------------
PvTPS62    331 RGRIIMTMFLANLTMLDDIYDSYGTPKECEIFTKCIE-----------------------
PvTPS08    352 RSRIILTMVIAIVTLLDDIYDSYATPEECEILTKCIE-----------------------
PvTPS36    352 RSRIILTMIIAIVTLLDDIYDSYATPEECEILTKCIK-----------------------
PvTPS81    355 RSRIILTMIIAIVTLLDDIYDSYATPEECEILTKCIK-----------------------
PvTPS18    254 LSRVILTKMTAFITILDDIIDTYSTTEEAMLLAKAIYRF---------------------
PvTPS16    279 LTRVITTKMTAYITILDDIMDTYSTTEEDKLLAEAIYSPTPPQIDLCIFNRLQICKNPAM
PvTPS109   319 LTRVITTKMTAYITILDDIMDTYSTTEEAILLAEAIYRW---------------------
PvTPS69    285 LTRVITTKMTAYITILDDIMDTYSTTEEAMLLAEAIYRW---------------------
PvTPS94    282 HSRIILTKITSLVTILDDMFDTYGTTEECIKFAEAINRW---------------------
PvTPS17    285 HSRIILAKMMGFITILDDFIDTYATTEESMQLAEAVFRW---------------------
PvTPS20    285 HSRIILAKMTGFITILDDFIDTYATTEESMQLAEAVFRW---------------------
PvTPS10    288 NARMMFAKFLTLSTFFDDIFDSYGTLDEVRQFNQAVQ-----------------------
PvTPS71    303 KARKLLAKFIATITPYDDTFDNYGIWEELQPFADVMQ-----------------------
PvTPS79    302 RARILYSKMTAFISLMDDTYDAHATFEECEFFNEAIQ-----------------------
PvTPS56    307 RARILFAKTFGMLSLMDDTYDVYATLEDCHILNEAIQ-----------------------
PvTPS05    297 RARMLFAKTFGMLSLMDDTYDVYATLEECHVLNEAIQ-----------------------
PvTPS09    235 RARIITTKYIILLSVLDDTYDIYATLDECRLLTIAFK-----------------------
PvTPS50    288 RARIITTKYIILLSVLDDTYDIYATLDECRLLTIAFK-----------------------
PvTPS54    298 RARMIL--IIAIIILTDDTYDVRATLEECRKFNEAIQ-----------------------
PvTPS33    326 RARMILAKIIAIIILTDDTYDVRATLEECRKFNEAIQ-----------------------
PvTPS53    343 RARMILAKIIAIIILADDTYDVRATLEECRKFNEAIQ-----------------------
PvTPS101   300 RARIILTKIFVLWTLLDVHFLI------------LSA-----------------------
PvTPS07    324 RARIILTKIFVLWTLLDDTFDTHANLEESQKLHQAIE-----------------------
PvTPS06    284 RARVILAKLFLLTSLLDDTYDVHATLEEARELNKAIE-----------------------
PvTPS55    289 RARMILVRLFILTSLLDDTYDDHATLEESRDLTKAIE-----------------------
PvTPS104   251 RARMILVRIFMLTSLLDDTYDEHATLGESRDLTLAIE-----------------------
[illegible]
```

```
PvTPS15   371 ------------------------------------------------------------
PvTPS106  324 ------------------------------------------------------------
PvTPS13   334 ------------------------------------------------------------
PvTPS02   323 ------------------------------------------------------------
PvTPS28   290 ------------------------------------------------------------
PvTPS27   329 ------------------------------------------------------------
PvTPS12   352 ------------------------------------------------------------
PvTPS73   322 ------------------------------------------------------------
PvTPS52   365 ------------------------------------------------------------
PvTPS01   321 ----------------------------------------------------------AI
PvTPS85   326 -------------------------------------------------------KGWAS
PvTPS19   329 ----------------------------------------------------------SL
PvTPS14   326 ----------------------------------------------------------SL
PvTPS11   328 ----------------------------------------------------------SL
PvTPS26   369 ------------------------------------------------------VLRTHV
PvTPS04   378 ------------------------------------------------------------
PvTPS83   378 ------------------------------------------------------------
PvTPS03   376 ------------------------------------------------------------
PvTPS62   368 ------------------------------------------------------------
PvTPS08   389 ------------------------------------------------------------
PvTPS36   389 ------------------------------------------------------------
PvTPS81   392 ------------------------------------------------------------
PvTPS18   293 ------------------------------------------------------------
PvTPS16   339 VSTISIGSIFAYLPTDSEGGGAEGRGGGGADDCETVAAPLQIILKIVLGFFQYHLVGSHI
PvTPS109  358 ------------------------------------------------------------
PvTPS69   324 ------------------------------------------------------------
PvTPS94   321 ------------------------------------------------------------
PvTPS17   324 ------------------------------------------------------------
PvTPS20   324 ------------------------------------------------------------
PvTPS10   325 ------------------------------------------------------------
PvTPS71   340 ------------------------------------------------------------
PvTPS79   339 ------------------------------------------------------------
PvTPS56   344 ------------------------------------------------------------
PvTPS05   334 ------------------------------------------------------------
PvTPS09   272 ------------------------------------------------------------
PvTPS50   325 ------------------------------------------------------------
PvTPS54   333 ------------------------------------------------------------
PvTPS33   363 ------------------------------------------------------------
PvTPS53   380 ------------------------------------------------------------
PvTPS101  325 ------------------------------------------------------------
PvTPS07   361 ------------------------------------------------------------
PvTPS06   321 ------------------------------------------------------------
PvTPS55   326 ------------------------------------------------------------
PvTPS104  288 ------------------------------------------------------------
[illegible]
```

```
PvTPS15   371 -MWDEHHKIEFYSEHVEIVFSAIYILVTRLGEKASLLQDR-------DVTKHLVEIW---
PvTPS106  324 -MWNTAAP-DSLPSCMRSCYDALYTITNEIADMAQKEHGF-------NPVNHLRKAW---
PvTPS13   334 -MWNTAVA-DSLPSGMRSCYKAIYNTTNEIADMVEVEHGF-------NPVNHLRNAW---
PvTPS02   323 -MWNTAVA-DSLPSGMRSCYKAIYNTTNEIADMVEVEHGF-------NPVNHLRNAW---
PvTPS28   290 -IWNTAAA-DSLPSCMRSCYKALYAITNEIAHRRYGRNGAWDEPCQSSQKSSMLLTW---
PvTPS27   329 -MWNTAAA-DSLPSCMRSCYKALYTITNEIADTAEKEHGM-------NPVNHLRKAW---
PvTPS12   352 -RWDENPC-EKLPEYMKELYSVIYDTTNEVAENILKIHGC-------SMHSVLGKAW---
PvTPS73   322 -RWEATAS-EQLPEYMKGIYFTIFNFSNVVAEHVQRTCGC-------DVRFLLKKGW---
PvTPS52   365 -RWEATAT-ELLPEYMKGIYLTIFNFSNEVAEHVQRTCGC-------DVRFLLKKVW---
PvTPS01   323 ERWDEQTT-EEFPAHLKPLLIGILDTTNKIEEELKLQKNR-------HA-EVVKKLV---
PvTPS85   331 ARWDKQSS-EKLTAYMKALFIFMLNTISDIMEELKLQKNK-------HA-EFVKELF---
PvTPS19   331 ERWDKQAA-EKMPAYMKDLFIFILNTINDIMEELKLQKNN-------HA-EFVKELF---
PvTPS14   328 ERWDEQAA-EKLPAYMKDLFIFTLNTINDIMEELKLRKNK-------HA-EFVKELF---
PvTPS11   330 ERWDKQAA-EKLPAYMKDLFIFTLNTINDIMGELKLQKNK-------HA-EFIKELF---
PvTPS26   375 SMWDTKFG-DGLPEGMKHALGKIFDSYETMDHELARDEKY-------RM-PYLKNLT---
PvTPS04   378 -RWDTKFA-DGLPECMKHALGKILDSFETIDHELAPEEKY-------RM-RYLKNFT---
PvTPS83   378 -RWDTKFG-DGLPECMKHALGKIFDSYEAMDHELARDEKY-------RM-AYLKNFT---
PvTPS03   376 -RWDTKFG-DGLPECMKHALGKIFDSYEAMDHELARDEKY-------RM-AYLKNFT---
PvTPS62   368 -SWYTNGA-HDLPEAMKFALRQTLDTYETIANMLHQEEKY-------RM-SYLINFT---
PvTPS08   389 -SWDAKAA-HDLPECIKFALGKVLDSFQNIENMLHQDEKY-------RM-SYLKYFT---
PvTPS36   389 -SWDAKAA-HDLPECIKFALGKILDSFQTIENMLHQEEKY-------RM-SYLKYFT---
PvTPS81   392 -SWDAKAA-HDLPECIKFALGKILDSFQTIENMLHQEEKY-------RM-SYLKYFT---
PvTPS18   293 ---NEDAT-ELLPDYMKDFYLFLLKTLDSCEDELGPNKRY-------RV-FYLKEMAKVH
PvTPS16   399 DSRIQNAA-EILPEYMKDFYLHLLKTFDTCEDELGPNKSF-------RV-FYLKELL---
PvTPS109  358 ---EENAS-ELLPEYVKDLYLHLLKTFNSCEDELGPNKSF-------RV-FYLKELL---
PvTPS69   324 ---EENAA-ELLPEYMKDLYLHLLKTFDSCEDELGPNKSF-------RV-FYLKELL---
PvTPS94   321 ---NESAV-PLLPEYMKGFYLFLLETFYSFEDELGPEKSY-------RV-LYLKEAM---
PvTPS17   324 ---DKDAI-TLLPEYTRDFYLFLLKTFCSFEEELGTGKSY-------RV-FYLKKAL---
PvTPS20   324 ---DKDAI-TLLPEYTRDFYLFLLKTFCSFEEELGTGKSY-------RV-FYLKKAL---
PvTPS10   325 -SWDEEAA-RKIGNCYAYVLSYFSDTYEAFVAD--NGASL-------MGVDYVKEAM---
PvTPS71   340 -RWDMKEV-EKLAIDFARFMFG---TMIEIENALPKDTGR-------RNVNFIR------
PvTPS79   339 -RWDESAA-SILPEYLRVFYIKILSTFKEFENILDPSEKY-------RV-GYVQTAF---
PvTPS56   344 -RWDESAV-STLPQYMRRFYINLLKTFQESEDSLQPHEKY-------RV-SYAKKAF---
PvTPS05   334 -RWDESAV-STLPEYMKKFYINLLKTFQECEDSLQPDEKY-------RV-SYAKKAF---
PvTPS09   272 -RWDKDSV-DMLPEYIRSFYLRFINSCNEIESELEPSEKF-------R--------T---
PvTPS50   325 -RWDKDAV-DMLPEYIKSFYLKFINSCNEIESELEPSEKF-------RV-SHFKSQT---
PvTPS54   333 -RWEESAI-TLLPDYLKKLYLKLMNIFKEFEDELKPNEKY-------RV-AFIRKAF---
PvTPS33   363 -RWEESAI-TLLPDYLKKLYLKLMNIFKEFEDELKPNEKY-------RV-AFVRKGF---
PvTPS53   380 -RWEESAI-TLLPDYLKKLYLKLTNIFKEFEDELEPNEKY-------RV-AFIRKAF---
PvTPS101  325 -KWDESAA-CLLPDYLTKFFLKLISNFREFDNELGPHEKY-------RS-AYNKKAF---
PvTPS07   361 -RWDESAA-CLLPDYLKKFFLKLISNFREFDNELGPHEKY-------RS-AYNKKAF---
PvTPS06   321 -RWDYGDV-SFLPEYLKKFFVKVVSNFSEFEDELEPHEKY-------RN-VYNRKGF---
PvTPS55   326 -RWDENNI-SFLPEYMKKFFLKVTRNFEEFEDELEPHEKY-------RV-AYVRKAF---
PvTPS104  288 -RWDENDI-SFLPEYMKKFFLKVLRNFEELR-----------------------------
```

```
PvTPS15    420 --------------------------LDLLKSMMTEVEWRINKYIPT-TEEYITNAALTFA
PvTPS106   372 --------------------------AVLFDGFMAESKWLATKQRAPGAEDYLRNGVVT-S
PvTPS13    382 --------------------------AALFDGFMVEARWLATD-EAPTAEDYLRNGVVT-S
PvTPS02    371 --------------------------AALFDGFMVEARWLATD-EAPTAEDYLRNGVVT-S
PvTPS28    345 --------------------------AELFDGFMVESKWLAAG-LVPAAEDYLRNGVVT-S
PvTPS27    377 --------------------------AELFDGFMVESKWLAAC-LVPAAEDYLRNGVVT-S
PvTPS12    400 --------------------------HDISVSFLLEAKWHHG-SYRPTLREYLDNGVVS-C
PvTPS73    370 --------------------------HDLCKAFLLEAKWHYYSNHKPTLQEYLDNGWMS-V
PvTPS52    413 --------------------------HDLCKAFLLEAKWHYYSNHKPTLQEYLDNGWMS-V
PvTPS01    371 --------------------------ICTAKFYHAEVNWRDEHYVPATVDEHLEKSLRS-S
PvTPS85    379 --------------------------IDMAKRYGAERKWRDEHYVPAKVKEHLQISVGS-S
PvTPS19    379 --------------------------IDTVKRYGAERKWCDERYVPAKIEEHLQISVAS-S
PvTPS14    376 --------------------------IDAVKRYGAERKWRDERYVPAKIEEHLQISVAS-R
PvTPS11    378 --------------------------VDAVKCYGTERKWSDERYVPAKIEEHLQISVAS-S
PvTPS26    423 --------------------------IDVIRGYNKEVKMCEEGYIPRTVNEHLQQR-----
PvTPS04    425 --------------------------IDLVRGYNAEVKMRDEGYIPRTVNEHLQVSLRT-G
PvTPS83    425 --------------------------IDLIRGYNKEVKMREEGYIPRTVNEHLQVSLRT-G
PvTPS03    423 --------------------------IDLIRGYNKEVKMREEGYIPRTVNEHLQVSLRT-G
PvTPS62    415 --------------------------KDIVRAYNMEAKMLEEGYIPKSVEEHLKVSLRT-G
PvTPS08    436 --------------------------EDLVRGFSREVKMLEKGYIPKSVEEHLQVSMRT-G
PvTPS36    436 --------------------------EDLVRGYNMEVNMLEEGYIPKFVEEHLQVSMRT-G
PvTPS81    439 --------------------------EDLVRGYNMEVNMLEEGYIPKFVEEHLQVSMRT-G
PvTPS18    341 MYYYLRGSTTKEKKIRILYQSYCLIKILVRGYSQEIQWRDEHYVPETINEHLKISGVT-I
PvTPS16    447 --------------------------KVLVRANSQEIKWRDEHYVPKTINEHLEISRAT-V
PvTPS109   403 --------------------------KLLVRANSQEIKWRDEHYVPKTINEHLEISRAT-V
PvTPS69    369 --------------------------KVLVRANCQEIKWRDEHYVPKTINEHLEISRAT-V
PvTPS94    366 --------------------------KRLVQQYSKEVEWRDEDYVPETMCEHLQVSMES-I
PvTPS17    369 --------------------------KQLVQAYIEELKWRDENYIPETLSEHLGLSMRS-S
PvTPS20    369 --------------------------KQLVQAYIEELKWRDENYIPETLSEHLGLSMRS-S
PvTPS10    371 --------------------------KEISSCMLKELVWREERQVPT-VHAYLTQAAVISV
PvTPS71    382 -------------------------------------DIRDNKYIPS-LEEHLKVTLVT-C
PvTPS79    386 --------------------------KLLSKYYLEEAKWCNEKYIPS-FKDQIEVSSMS-S
PvTPS56    391 --------------------------QLSSKYYLDEAKWCSEKYAPS-FEEHVEVSVMS-S
PvTPS05    381 --------------------------QLSSKYYLDEAKWSSEKYAPS-FEEHVEVSVMS-S
PvTPS09    312 --------------------------EIMVDAYLQEASWYAQDMFPE-VDIRKELSVVRSS
PvTPS50    372 --------------------------EIMVDAYLQEASWYAQDMFPE-VDIRKELSVVKSS
PvTPS54    380 --------------------------QVLSSNYLQEAEWCHGGYRPR-FKDQVKVSTVC-S
PvTPS33    410 --------------------------QVLSSNYLQEAEWFHGGCKPR-FKDQVKVSTVC-S
PvTPS53    427 --------------------------QVLSSNYLQEAEWCHSGYKPR-FKDQVKVSTVC-S
PvTPS101   372 --------------------------QKLSSYYLQESEWFHKNHIPS-FKDQMDVSVMT-G
PvTPS07    408 --------------------------QKLSSYYLQESEWFHKNHMPS-FKDQMDVSVMT-G
PvTPS06    368 --------------------------QTLSKYYLQEAEWFHQGFTPS-FKEQVSVSVIT-A
PvTPS55    373 --------------------------QLISKSYLQEAEWSHHEYIPS-FKDHVNVSAIS-A
PvTPS104   317 -------------------------------------------------------------
```

```
PvTPS15   454 LGPIV-LPALYFVGPKIPE------SAVKDLEYNELFRLMSTCGRLLNDVQTYEREYR-E
PvTPS106  406 GVPLIFAHLFYLLGQDHVASTNEDAAKPLSDDIPPAISCLAKILRLWDDLGSAKDEAQ-E
PvTPS13   415 GVPLTFAHIFAMLGYDKS---NEAGAKLADDHIPSVISCPAKILRLWDDLGSAEDEAQ-E
PvTPS02   404 GVPLTFAHIFAMLGYDKS---NEAGAKLAYDHIPSVISCPAKILRLWDDLGSAEDEAQ-E
PvTPS28   378 GMPLTLAHVLFLLGQDHAAYS---DVTKLTDHIPLAISCPGKILRLWDDMGSAEDEAQ-E
PvTPS27   410 GMPLALAHVLFLLGHDHAAFG---DAARFTDHIPPAISCPGKILRLWDDMGSAEDEAQ-E
PvTPS12   433 SAPLLLLHAFPMLNSELNAR----TFSLI-QSNPRLLQSASLVLRLCNDSATHSAELQ-R
PvTPS73   404 SGPLMLLHAFPMLNEAITQK----SIEQLESHYPKLVQMVSKIFRLCNDLAKHSV--R-A
PvTPS52   447 SGPLMLLHAFPMLNEAITQN----SIEQLESHYPKLVQMVSKIFRLCNDSATHSEELK-R
PvTPS01   405 VCMQIIIGVLISLRD-C-RE----DDVNWAFTFPKLIRGVSVVGRVGNDIVSDEREQA-S
PvTPS85   413 ASMHIVNVSFILMGD-ITTR----EAIEWAFSYPEMIRA---------------REQV-S
PvTPS19   413 ACMHLANITFVLMGD-VTTG----EAIEWAFSYPEMIRAACIVARVCNDIMSHEREQA-S
PvTPS14   410 GCMHLANITFVLMGD-VTTR----LAIEWAFSFPEMIRAACTVGRICNDIMSHEREQV-S
PvTPS11   412 ACMHLANITFVLMGD-VTTR----EAIEWAFSFPEMIRAACTVGRICNDIMSHEREQV-S
PvTPS26   453 -------------------R----ILFDWVSSMPKIVEDLGIIVRLLDDLQSYEREQL-T
PvTPS04   459 ACHLLACASFVGMDD-IATK----DSFDWVSTMPKIVKALCIILRLLDDLQTYEREKM-T
PvTPS83   459 ACHLLACASFVGMDD-IATK----DSFDWVSTMPKIVKDLCIILRLLDDLQSYEREQL-T
PvTPS03   457 ACHLLACASFVGMDD-IATK----DSFDWVSTMPKIVKDLCIILRLLDDLQSYEREQL-T
PvTPS62   449 GCPILPCASFIGMHD-IATK----EFFDWVASLPNMVQALSVILRLVDDLQTYEREKL-N
PvTPS08   470 GCPILSCASFVGMND-IATK----DCFDWISHVPNMVQALARVLRLLDDLQSYEREQL-I
PvTPS36   470 GCPLLSCASLVGMND-IATR----DCFDWVSRVPNMVRALARILRLLDDLQTYEREQL-I
PvTPS81   473 GCPLLSCASLVGMND-IATR----DCFDWVSRVPNMVRALARILRLLDDLQTYEREQL-I
PvTPS18   400 GAFQLVCSSFVGMGD-IITK----EVLDWLLAFPELLNCFPTFARLSNDIASTEREQT-R
PvTPS16   481 GGFQVACSSFVGMGD-VITK----ENLDWLLTYPELLKYFSTIARLSNDIKSTEREQI-G
PvTPS109  437 GGFQVACSSFVGMGD-IITK----EILDWLLTYPELLKCFSTIARLSNDIKSTEREQI-G
PvTPS69   403 GGFQVACSSFVGMGDDIITK----EILDWLLTYPELLKCFSIIARLSNDIKSTEREQI-G
PvTPS94   400 GSVALACAAYVGMGD-VITK----GTLEWVLSYPQFLTSFGVFVRLSNDLVSTKREQT-A
PvTPS17   403 GGSPILCASLVGMGE-IVTR----EALDWFLSYPQLVRSFDTFVRLSDDMASTEREQK-G
PvTPS20   403 GGAQILCSSLVGMGE-IVTR----ETLDWFLSYPQLVRSFDTFVRLSDDMASTEREQK-G
PvTPS10   405 QYVPAAVTALVGMNA---KD----EVLSWAGSYPKIIEIAATMCRLMDDVAGHENEKEDR
PvTPS71   404 FYWGICCAAFVVFEE-NVTE----ELLKWMSKFPQIVKDSCIISRLMDDIVAHEFETE-R
PvTPS79   419 SIPVLALAALMAAGD-EATN----EAVEWASGIPDAVHACGEIGRLLNDISAFKKGRKNK
PvTPS56   424 GFPTLAVVLLMGAGD-LATR----EAFEWAIGEPAVVSASGEVARFLNDIASYRKGKN-K
PvTPS05   414 GFPTLAVVLLMGAGD-LATR----EAFEWAIGVPAVVSASGEVARFLNDIASYRKGKN-K
PvTPS09   346 GYPELTCVSFIGMGE-VATK----EAFDWVTSIPKIVRASAEIYRFTDDIYSSEREHR-V
PvTPS50   406 GYPELSCASFIGMGE-VATK----EAFDWVTSIPKIVRASAEICRFTDDIYSIEREHR-V
PvTPS54   413 GAPFAAVGLLVGMGDDVATK----EALEWATSCTDAVKAFADVTRFMNDLCSFKRGKN-K
PvTPS33   443 GAPFASVGLLDGMGDD-------------------AVKAFADVTRFMNDLCSFKRGKN-K
PvTPS53   460 GAPFAAVGLLVGMGDDVATK----EALEWATSCTDAVKAFADVTRFMNDLCSFKRGKN-K
PvTPS101  405 GAQMACVGILFGMDD-VAP-----DAFEWAIGCSDSAKTVGAITRYANDLAAFKNGGN-K
PvTPS07   441 GAQMACVGILFGMDD-VAP-----DAFEWAIGCSDSAKTVGAITRYVNDLAAFKNGGN-K
PvTPS06   401 GAQMLSIGLLVGMGD-VATK----EAFEWVIGNSDAIWACGEVSRFMDDMSAFKNGRN-K
PvTPS55   406 GGQVMCVGSLVGMGD-VATK----EAFEWAIGHTDAIRASGEVSRFMDDMADFKRGRD-K
PvTPS104  317 --TMMCVGSLVGMGD-VATK----EAFEWAIGSTDAIRASGEVSRFMDDMADFKRGRN-K
```

```
PvTPS15    506 GKVNSVSLLIHQSGGSLSIAEARREIQKPIDTCRRDLLGLVLRKEG---------------
PvTPS106   465 GLDGSYRDLYLMENPSCTPADAEEYMRRLIKREWEELNRECFSRRT---------------
PvTPS13    471 GLDGSYRDFYLMENPSCTPGDAEAHMRRLIAREWEELNRECFSRRT---------------
PvTPS02    460 GLDGSYRDFYLMENPSCTPGDAEAHMRRLIAREWEELNRECFSRRT---------------
PvTPS28    434 GLDGSYRDLYLMENPSCTPADAEEHMRRMIKREWEELNRECFSRRI---------------
PvTPS27    466 GLDGSYRDSYLMENPSCTPADAEEHMRRLIAREWEELNRECFSRTT---------------
PvTPS12    487 GDAPSSIAIH-MSESGATEQDSRKAMEDLIMEAWKTINQEAFGSCK---------------
PvTPS73    457 LDAPSSIAIY-MFENRTMETDARRAMRDLTMETWKIVIQDVYNNCQ---------------
PvTPS52    502 GDAPSSVAIY-MFENRAMETNARRAMRDLTMETWKIVNQDVYDKCQ---------------
PvTPS01    458 EHVVSTVQTC-MKQYGITAEQANEKLRVIIEEAWMDIVQEYLDQ-----------------
PvTPS85    452 KHVASTVQTC-MKEYGITVDEANE-------------------------------------
PvTPS19    467 KHVASTVQTC-MKEYGMTVHQAYEKLGDLIDEAWMDIVQGCLDQ-----------------
PvTPS14    464 KHVASTVETC-MKEYGMTVHQAYEKLRALIDEAWMDIVQGCLDQ-----------------
PvTPS11    466 KHVASTVETC-MKEYGMTVHQAYEKLRALIDEAWMDIVQGCLDQ-----------------
PvTPS26    489 PHVASTIKSY-MKEHSVSIQIARKKIEELKEDTWKDFNLEWLNPNN---------------
PvTPS04    513 PHVASTIKSY-MKEHSVSMEIARKKIAELKEDTWKDFNREWLNPDN---------------
PvTPS83    513 AHVASTIKSY-MKEHNVSIQIARKKIEELKEDTWKDFNHEWLNPDN---------------
PvTPS03    511 AHVASTIKSY-MKEHNVSIQIARKKIEELKEDTWKDFNHEWLNPDN---------------
PvTPS62    503 PHFASTIDSY-MKEHNVSIEVAREKIHILKEKSWKDFNSEWLNPDN---------------
PvTPS08    524 PHVASTIDSY-MKEHNVSIEVAREKIYKLKEESWKDFNSEWLNPDN---------------
PvTPS36    524 PHVASTIDSY-MKEHNVSIEVAREKIHTLKEDSWKDFNSEWLNPDN---------------
PvTPS81    527 PHVASTIDSY-MKEHNVSIEVAREKIHTLKEDSWKDFNSEWLNPDN---------------
PvTPS18    454 GHHASTIQCY-MLQHETTMHDACEKIKELIEDSWKDMVKLYLTPT----------------
PvTPS16    535 AHHASTVECY-MLQHRTTMSDAFERIKDLIEDAWKDMMKLFLTPT----------------
PvTPS109   491 AHHASTVECY-MLQHRTTMNDAYERIKDLIEDAWKDMMKLFLTPT----------------
PvTPS69    458 AHHASTIECY-MLQHRTTMNDAYERIKVLIEDAWKDMMKLFLTPT----------------
PvTPS94    454 DHSASTVHCY-MKEHGTTMNDACEKIKELTEDLWKDMLEQCLALK----------------
PvTPS17    457 DHSVSTVQCY-MKEHGATMHEACKRLKELTEDLWKGMVQHHLAST----------------
PvTPS20    457 DHSVSTVQCY-MKEHGTTMHEACKRLQELTEDLWKDMVQRHLAST----------------
PvTPS10    458 SRCFTAVDCY-VNEHGATVQQAKKALRGFLEEHWRRINQEFLSNVT---------------
PvTPS71    458 NNVATAVTCY-MKEYKTTKEEASEVLWGSVENAWKSMNNEYLTWTS---------------
PvTPS79    474 NDVASSLECY-MKEYGTRGEEAAAALSAMVEHAWRRINKACMEIDR---------------
PvTPS56    478 KDALSSVECY-ARERGVSGEEAAAAIAGMAEHAWRTINRSCMEVGG---------------
PvTPS05    468 KDALSSVECY-ARERGVSGEEAAAAIAGMAEHAWRTINRSCMEVGG---------------
PvTPS09    400 GQCANTFDCY-MKQYNATSEETKERFLSWIEDAWRTIKEECIVET----------------
PvTPS50    460 GQCASTFDCY-MKQYNATSEETKERFLSWIEDAWRTINEECIVET----------------
PvTPS54    468 NDVDSSVECY-ISEHGVTADVACAKIDSLIEDAWKTANRARIEQNELLPARPGHGVHGPT
PvTPS33    483 NDVDSSVECY-ICEHGVTADVACAKIDSLVEDAWKTANRARIEHNEPRP------------
PvTPS53    515 NDVDSSVECY-ISEHGVTADVACAKIDSLVEDAWKTANRARIEHNELLP------------
PvTPS101   458 MDTANSVECY-IKEHNVTSEVALAKISDLVEHEWKNTNEARFKNR----------------
PvTPS07    494 MDTANSVECY-IKEHNVTSEVALAKISDLVEHEWKTTNEARFKNR----------------
PvTPS06    455 LDVPSTVECY-IKEHNVSSDVALTKISSFVEDAWKTINQAPFKYP----------------
PvTPS55    460 MDVATSVECY-MKEHNVTSEVAVAKIGSFVDVAWKTINQALFDHRS---------------
PvTPS104   369 MDVATSVECY-MKEHNVTGEVALAKIGSFVDEAWKTLNQALFDHRS---------------
[illegible]
```

```
PvTPS15   552 -VVPRPCKE-LFWKMCKVCYFFYS-RGDAFSSPDEKARE-VDAVVNLP--LLLKGSSNVS
PvTPS106  511 --FSGSFVQ-ACFNAAKMVSVMYSY--DKEQKLPVLE-DYMRMLLL--------------
PvTPS13   517 --FSSRFTQ-ASLNVARMVSVMYSY--DKEQRLLVLE-DYATMLLL--------------
PvTPS02   506 --FSSRCNR-PV------------------------------------------------
PvTPS28   480 --FSSSFMQ-ACLNAARMVSVMYSY--DKEQRLLVLE-DYARMLLL--------------
PvTPS27   512 --FSSSFMQ-ACLNAARMVSVMYSY--DKEQRLLVLE-DYAKMLLL--------------
PvTPS12   532 --FARPFAK-ACVNLARISQCVYH-RGDGFGEPSDVKRKQINDLFLEPAYS---------
PvTPS73   502 --YPPSFAN-ACVNMARISHCIYQ-GGDGVSAPDDGKRKEISELFLEP------------
PvTPS52   547 --YPPSFAN-ACVNMARISHCIYQ-GGDGLGAPDDGKRMEISELFLEPLKAAPDGGRH--
PvTPS01   501 -KRPREFLE-KSVDVARTMDFFYK-RDDAYTLPLSIK-DTITLMYVNPCEL---------
PvTPS85   475 ------SLE-KAVNVARVMDNMYK-RDDAYTHPYSLK-DTITSMYVNSA-----------
PvTPS19   510 -YYPMEHLE-KVVNIARGMDHMYK-RDDAYTHPHSLK-DTITSMYVNSV-----------
PvTPS14   507 -PYPMEILE-KVVNIARTMDKMYK-RDDAYTDPYSLK-DTITSMYVNPV-----------
PvTPS11   509 -PYPMEILE-KVVNIARTMDKMYK-RDDAYTDPYSLK-DTITSMYVNSV-----------
PvTPS26   534 -EVPRQLLE-RIFNLTRTMDFIYN-QDDNFTNCQNLR-DTIHLLFVEPFAISL-------
PvTPS04   558 -SVPRQLLE-NIFNLTRTMEFMYN-LDDNFTNCQNLK-DTIHLLFVEPFAISI-------
PvTPS83   558 -GVPRQLLE-SIFNMTRTMEFMYN-QDDNFTNSQNLK-DTIHLLFVEPFAISF-------
PvTPS03   556 -GVPRQLLE-SIFNMTRTMEFMYN-QDDNFTNSQNLK-DTIHLLFVEPFAISI-------
PvTPS62   548 -TYPKQLLE-RIFNLTRTVEFIYN-KDDNFTNCGNIR-DTIQSLLVEPFMVIL-------
PvTPS08   569 -TYPKQVLE-RIFNLTRTMEFMYN-QEDNFTNCPNLK-DTIYLLFAEPFAN---------
PvTPS36   569 -TYPKQVLE-RIFNLTRTMEFMYN-QEDNFTNCPNLK-DTINLLFVEPFTELI-------
PvTPS81   572 -TYPKQVLE-RIFNLTRTMEFMYN-QEDNFTNCPNLK-DTINLLFVEPFTELI-------
PvTPS18   498 -EQMKVVAQ-TVVDFARTGEYMYK-KTDGFTFSHTIK-DMVALLYVEPILF---------
PvTPS16   579 -EQPKLIAK-TIVDFARTVDYIYK-KSDAFTFSHTIK-DMITMLYVDPTLF---------
PvTPS109  535 -GQPKLIAK-TVVDFARTVDYIYK-KTDAFTFSHTIK-DMITMLYVEPTLF---------
PvTPS69   502 -EQPKLIAK-TVVDFARTADYIYK-KTDAFTFSHTIK-DKITMLYVEPILF---------
PvTPS94   498 -GLPKVVPR-TVFDFSRTTDNMYK-NCDAFTSSQALK-QMIELLFVEPIPE---------
PvTPS17   501 -EQTEIVSC-MVLNLARTGNYMYQNNVDKFTSSHTIK-EAIRRLFVEPIPV---------
PvTPS20   501 -EQTEIVSR-MVLNLARTGNYMYQNNVDKFTSSHTIK-DAIRRLFVEPIPV---------
PvTPS10   503 --VPVPLLT-VLIDIVRIMDSMYID-VDAYSKCSKLA-DPIHKLLNECVHH---------
PvTPS71   503 --IPS-KLLIRVINLARMMETMYKN-IDGYTDSKILK-EWISVLLDQPIPF---------
PvTPS79   519 --GLLPAVKLAVINLGRSNEIVYCRGNDAYTFTGDLE-GLVTSLFLKPVPI---------
PvTPS56   523 --ALLPAARL-VVNLTKTLEVIYLGGRDAYTFAGDLR-DLVVSLFLNGPAA---------
PvTPS05   513 --ALLPAARL-VVNLTKTLEVIYLGGRDAYTFAGDLR-DLVVSLFLNGPAI---------
PvTPS09   444 --VPRPLIE-RVVNWAKIMHVIYTHCDNGYSMCENLK-DYIAVLFEESLPF---------
PvTPS50   504 --VPRPLIE-RVANWAKIMHVIYTHCDNGYNMCENLK-DYIAVLLEEPLPF---------
PvTPS54   527 RSDPVIAVQ-RVVNITTSMPLMYGDKKDAFTFSDGLK-GVIKRLFLEPVLL---------
PvTPS33   531 ------AVQ-RVVNIMTSMPLMYGDKKDAFTFSDGLK-GVIKRLFLEPVLL---------
PvTPS53   563 ------AVQ-RVVNITTSMPLMYGDKKDAFTFSDGLK-GVIKHLFLEPVLL---------
PvTPS101  502 --ELLSVVQ-RVSNCAMCAMFYCHGMRDLYTNSKDII-GAIESHFVNPISL---------
PvTPS07   538 --ELLSVVQ-RVSNCAMCAMFFCHGMRDLYTNSKDLI-GTIESHFVNPISL---------
PvTPS06   499 --ALLPVVR-RVASLAKSMTLLFLDKRDAYTYSKDFK-KTLESHFVKHI-----------
PvTPS55   505 --PPLPVLQ-RVANFAMSIMIIFLDQRDGYTNSKEFK-ETLESQFVKHIPL---------
PvTPS104  414 --LPLPVLQ-RVTNFAMSIMLIFLDQRDGYTNSKEFK-DSGEPIRQAYSPLILEQQL---
```

```
PvTPS15    606 LPILWEGSNQHAWKMCKVCYFFYS-RGDAFSSPDEKARE-VDAVVNLP--LLLKGSSNVS
PvTPS106       ------------FNAAKMVSVMYSY--DKEQKLPVLE-DYMRMLLL--------------
PvTPS13        ------------LNVARMVSVMYSY--DKEQRLLVLE-DYATMLLL--------------
PvTPS02        ------------------------------------------------------------
PvTPS28        ------------LNAARMVSVMYSY--DKEQRLLVLE-DYARMLLL--------------
PvTPS27        ------------LNAARMVSVMYSY--DKEQRLLVLE-DYAKMLLL--------------
PvTPS12        ------------VNLARISQCVYH-RGDGFGEPSDVKRKQINDLFLEPAYS---------
PvTPS73        ------------VNMARISHCIYQ-GGDGVSAPDDGKRKEISELFLEP------------
PvTPS52        ------------VNMARISHCIYQ-GGDGLGAPDDGKRMEISELFLEPLKAAPDGGRH--
PvTPS01        ------------VDVARTMDFFYK-RDDAYTLPLSIK-DTITLMYVNPCEL---------
PvTPS85        ------------VNVARVMDNMYK-RDDAYTHPYSLK-DTITSMYVNSA-----------
PvTPS19        ------------VNIARGMDHMYK-RDDAYTHPHSLK-DTITSMYVNSV-----------
PvTPS14        ------------VNIARTMDKMYK-RDDAYTDPYSLK-DTITSMYVNPV-----------
PvTPS11        ------------VNIARTMDKMYK-RDDAYTDPYSLK-DTITSMYVNSV-----------
PvTPS26        ------------FNLTRTMDFIYN-QDDNFTNCQNLR-DTIHLLFVEPFAISL-------
PvTPS04        ------------FNLTRTMEFMYN-LDDNFTNCQNLK-DTIHLLFVEPFAISI-------
PvTPS83        ------------FNMTRTMEFMYN-QDDNFTNSQNLK-DTIHLLFVEPFAISF-------
PvTPS03        ------------FNMTRTMEFMYN-QDDNFTNSQNLK-DTIHLLFVEPFAISI-------
PvTPS62        ------------FNLTRTVEFIYN-KDDNFTNCGNIR-DTIQSLLVEPFMVIL-------
PvTPS08        ------------FNLTRTMEFMYN-QEDNFTNCPNLK-DTIYLLFAEPFAN---------
PvTPS36        ------------FNLTRTMEFMYN-QEDNFTNCPNLK-DTINLLFVEPFTELI-------
PvTPS81        ------------FNLTRTMEFMYN-QEDNFTNCPNLK-DTINLLFVEPFTELI-------
PvTPS18        ------------VDFARTGEYMYK-KTDGFTFSHTIK-DMVALLYVEPILF---------
PvTPS16        ------------VDFARTVDYIYK-KSDAFTFSHTIK-DMITMLYVDPTLF---------
PvTPS109       ------------VDFARTVDYIYK-KTDAFTFSHTIK-DMITMLYVEPTLF---------
PvTPS69        ------------VDFARTADYIYK-KTDAFTFSHTIK-DKITMLYVEPILF---------
PvTPS94        ------------FDFSRTTDNMYK-NCDAFTSSQALK-QMIELLFVEPIPE---------
PvTPS17        ------------LNLARTGNYMYQNNVDKFTSSHTIK-EAIRRLFVEPIPV---------
PvTPS20        ------------LNLARTGNYMYQNNVDKFTSSHTIK-DAIRRLFVEPIPV---------
PvTPS10        ------------IDIVRIMDSMYID-VDAYSKCSKLA-DPIHKLLNECVHH---------
PvTPS71        ------------INLARMMETMYKN-IDGYTDSKILK-EWISVLLDQPIPF---------
PvTPS79        ------------INLGRSNEIVYCRGNDAYTFTGDLE-GLVTSLFLKPVPI---------
PvTPS56        ------------VNLTKTLEVIYLGGRDAYTFAGDLR-DLVVSLFLNGPAA---------
PvTPS05        ------------VNLTKTLEVIYLGGRDAYTFAGDLR-DLVVSLFLNGPAI---------
PvTPS09        ------------VNWAKIMHVIYTHCDNGYSMCENLK-DYIAVLFEESLPF---------
PvTPS50        ------------ANWAKIMHVIYTHCDNGYNMCENLK-DYIAVLLEEPLPF---------
PvTPS54        ------------VNITTSMPLMYGDKKDAFTFSDGLK-GVIKRLFLEPVLL---------
PvTPS33        ------------VNIMTSMPLMYGDKKDAFTFSDGLK-GVIKRLFLEPVLL---------
PvTPS53        ------------VNITTSMPLMYGDKKDAFTFSDGLK-GVIKHLFLEPVLL---------
PvTPS101       ------------SNCAMCAMFYCHGMRDLYTNSKDII-GAIESHFVNPISL---------
PvTPS07        ------------SNCAMCAMFFCHGMRDLYTNSKDLI-GTIESHFVNPISL---------
PvTPS06        ------------ASLAKSMTLLFLDKRDAYTYSKDFK-KTLESHFVKHI-----------
PvTPS55        ------------ANFAMSIMIIFLDQRDGYTNSKEFK-ETLESQFVKHIPL---------
PvTPS104       ------------TNFAMSIMLIFLDQRDGYTNSKEFK-DSGEPIRQAYSPLILEQQL---
[illegible]
```

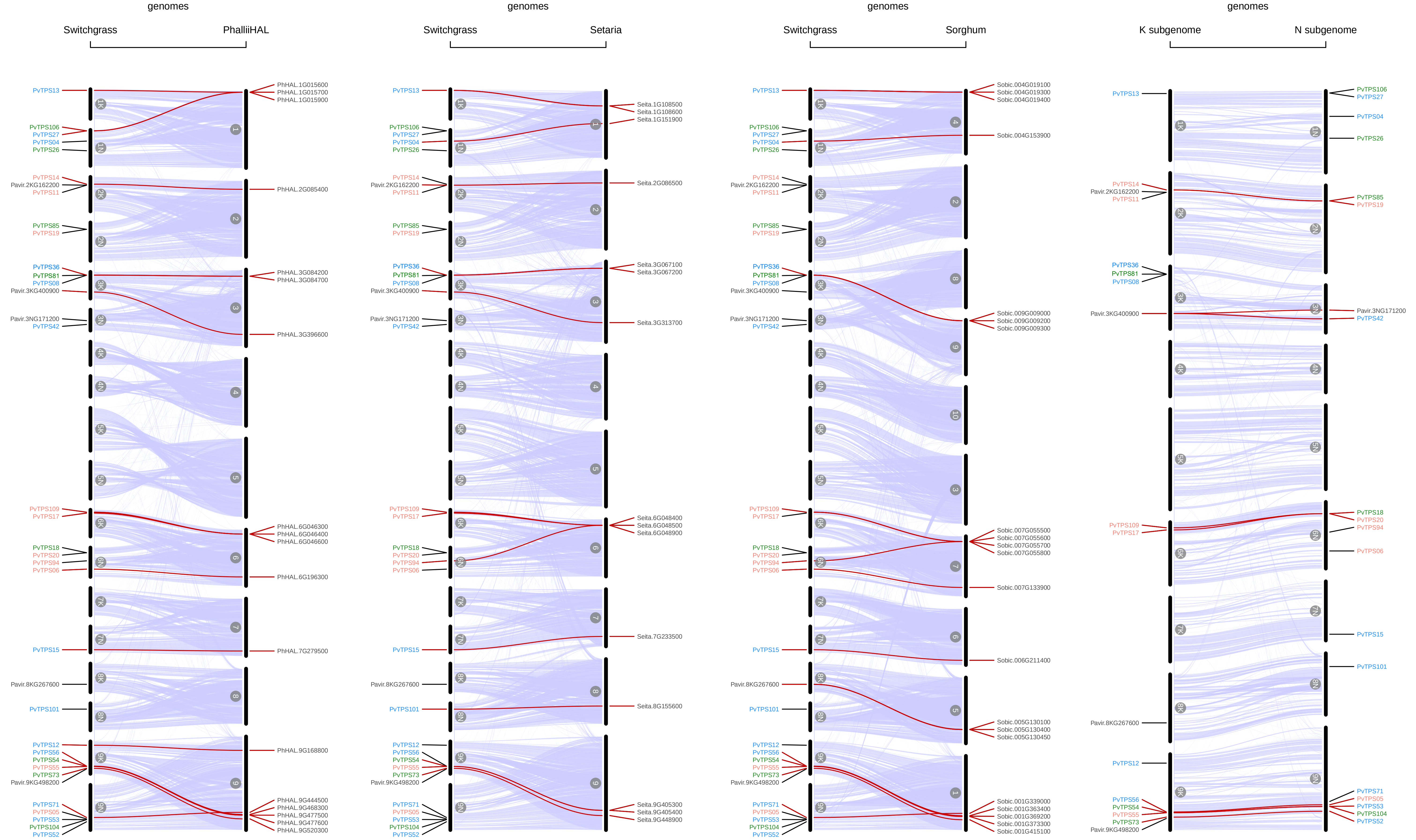

Supplementary Figure S3. Synteny and orthology of TPS gene family members between *P. virgatum* sub-genomes and between genomes of *P. virgatum*, *S. bicolor*, *S. italica*, and *P. hallii*. In each plot, the genomic positions of chromosomes are plotted along the x-axis. The scale is independent for each genome and chromosomes are ordered to maximize synteny with *P. virgatum*. Chromosome IDs are printed on the inside of each line segment. Syntenic blocks between each pair of (sub)genomes are presented as light blue polygons. Characterized *P. virgatum* mono-TPS and sesqui-TPS genes (labeled PvTPS, color code according to Fig. 3) and annotated TPS genes (labeled with the alpha-numeric Phytozome gene IDs) that occur in orthologous gene networks of any of the analyzed genomes are shown. The positions of genes are indicated by a straight line. A red line indicates that there is an ortholog in the alternative genome.

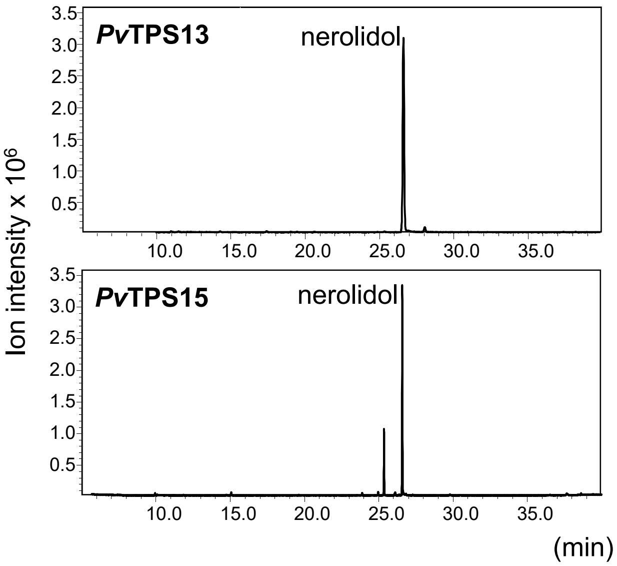

Supplementary Figure S4. SPME-GC-MS analysis of volatile products produced by recombinant *Pv*TPS13 and *Pv*TPS15 with (*E,E*)-FDP as a substrate.

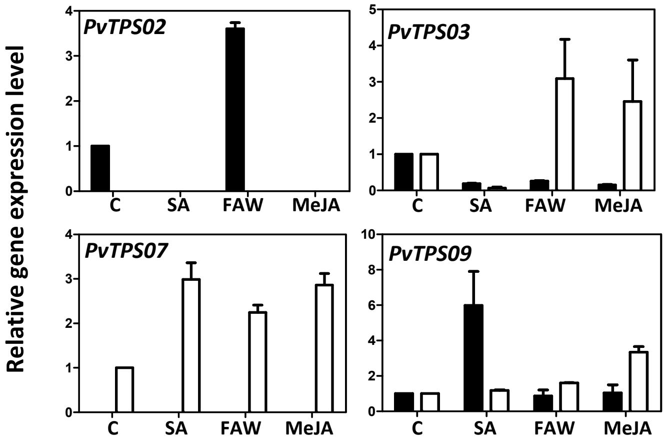

Supplementary Figure S5. Transcript levels of selected TPS genes induced less than 10 fold in leaves (black bars) and/or roots (white bars). Samples were analyzed in biological and technical triplicate and normalized to the expression of the control gene elongation factor 1a (*ELF1α*). Control (C) expression levels were scaled to 1 for comparison of treatment effects. SA: salicylic acid; FAW, fall armyworm; MeJA, methyl jasmonate.
